# Prioritization of microRNA biomarkers for a prospective evaluation in a cohort of myocardial infarction patients based on their mechanistic role using public datasets

**DOI:** 10.3389/fcvm.2022.981335

**Published:** 2022-11-03

**Authors:** Priyanka Venugopal, Melvin George, Sriram Damal Kandadai, Karthikeyan Balakrishnan, Chakradhara Rao S. Uppugunduri

**Affiliations:** ^1^Clinical Research Department, Hindu Mission Hospital, Chennai, India; ^2^Diabetology and Endocrinology, Hindu Mission Hospital, Chennai, India; ^3^Department of Cardiology, Hindu Mission Hospital, Chennai, India; ^4^CANSEARCH Research Platform in Pediatric Oncology and Hematology, Department of Pediatrics, Gynecology and Obstetrics, University of Geneva, Geneva, Switzerland

**Keywords:** myocardial infarction, microRNA, gene regulatory network, computational biology, association

## Abstract

**Background:**

MicroRNAs (miR) have proven to be promising biomarkers for several diseases due to their diverse functions, stability and tissue/organ-specific nature. Identification of new markers with high sensitivity and specificity will help in risk reduction in acute myocardial infarction (AMI) patients with chest pain and also prevent future adverse outcomes. Hence the aim of this study was to perform a detailed *in silico* analysis for identifying the mechanistic role of miRs involved in the pathogenesis/prognosis of AMI for prospective evaluation in AMI patients.

**Methods:**

miR profiling data was extracted from GSE148153 and GSE24591 datasets using the GEO2R gene expression omnibus repository and analyzed using limma algorithm. Differentially expressed miRs were obtained by comparing MI patients with corresponding controls after multiple testing corrections. Data mining for identifying candidate miRs from published literature was also performed. Target prediction and gene enrichment was done using standard bioinformatics tools. Disease specific analysis was performed to identify target genes specific for AMI using open targets platform. Protein-protein interaction and pathway analysis was done using STRING database and Cytoscape platform.

**Results and conclusion:**

The analysis revealed significant miRs like let-7b-5p, let-7c-5p, miR-4505, and miR-342-3p in important functions/pathways including phosphatidylinositol-3-kinase/AKT and the mammalian target of rapamycin, advanced glycation end products and its receptor and renin–angiotensin–aldosterone system by directly targeting angiotensin II receptor type 1, forkhead box protein O1, etc. With this approach we were able to prioritize the miR candidates for a prospective clinical association study in AMI patients of south Indian origin.

## Introduction

India has accounted the highest mortality rate of approximately 21% due to cardiovascular disease in 2010 and majority of the deaths was due to coronary artery disease (CAD). The most critical outcome of CAD is myocardial infarction (MI or acute MI) (1). Although there are several treatment strategies for MI, the amount of cardiac deaths and other enduring complications post-MI is still high. There are currently limited options for prognosticating patients with acute MI. MicroRNAs (miRs) are non-coding RNAs which functions in regulating gene expression by binding to the 3’-unttranslated region of the genes they target (2). They are expressed stably and are tissue-specific. Researchers are focusing on unraveling the key role and functions of miRs so as to prognosticate patients with acute MI (3).

Over the last decade, studies have reported several miRs which control key processes that contribute to the pathophysiological consequences of acute myocardial infarction (AMI) (4). The aberrant expression (over expression/down regulation) of miRs post-AMI activates certain downstream processes in a cell-specific manner (2). They mainly function *via* regulation of apoptotic, necrotic and autophagic cell death. Many miRs have been reported as ideal biomarkers (diagnosis and prognosis) and therapeutic targets in MI. Some of these include miR-1, miR-21, miR-30a, and miR-499 (5). However, prospective evaluation of the role of miRs in AMI and other cardiovascular events post-AMI remain unexplored. In this regard, the present study has aimed at prioritizing the miR candidates for a prospective evaluation in AMI patients of south Indian origin *via* exploring the existing datasets on miRs in MI setting. We also aim to explore relevance of the identified miRs in the pathogenesis of AMI.

## Methodology

### Microarray data from gene expression omnibus database

The miR profiling dataset for AMI was retrieved from GEO database using search terms “myocardial infarction’ AND “Homo sapiens” AND “microRNA” AND “serum” “non-coding RNA profiling by array” OR “non-coding RNA profiling by high-throughput sequencing” (performed in March, 2022). The search revealed 241 possible datasets which were further filtered to exclude other diseases, sample type, methylation and candidate miR assays. Two datasets (GSE148153 and GSE24591) were found to be appropriate for the study (6–10). GSE148153 dataset (7) (platform: GPL20712) comprised of 28 samples (healthy controls—7, fulminant myocarditis—16 and myocardial infarction—5), among which 5 healthy controls (controls) and 5 myocardial infarction samples (test) were analyzed. Peripheral blood samples were collected from controls and test group at the onset. Serum was separated from the blood samples and used for miR profiling. Of the 5 AMI patients, 4 were males and 1 was a female. GSE24591 (10) (GPL8227 platform) comprised of 4 first AMI (FAMI) patients and 3 sets of healthy controls (each set consisting of 42 blood donors pooled together). The pooled control group included equal number of males and females who were age stratified as 28–45, 45–60, and > 60 years of age. These participants were clinically tested and excluded from any cardiovascular disease/condition. Platelets obtained from the FAMI patients were collected within 6 h of the onset of symptoms. The characteristics of FAMI patients are shown in Table 1. Analysis was performed between healthy controls (controls) and myocardial infarction samples (test) using the limma algorithm (11). Parameters used for the analysis included Benjamini-Hochberg false discover rate correction, log transformation, data normalization and *p*-value cut-off of < 0.01.

**TABLE 1:** Baseline characteristics of FAMI (cases) patients derived from GSE24591 dataset (10).

Patient ID	Age/Gender	BMI	Sample type	Additional information
N.16	56/male	19.59	Platelets	cTnI < 0.2 pg/ml CRP—0.18 mg/dl IL6—2.55 ng/dl
N.26	52/male	29.07		cTnI—0.71 pg/ml CRP—1.07 mg/dl IL6—1.06 ng/dl
N.27	74/male	21.67		cTnI < 0.2 pg/ml CRP—1.37 mg/dl IL6—0.85 ng/dl
N.33	37/male	23.5		cTnI < 0.2 pg/ml CRP—11.40 mg/dl IL6—4.95 ng/dl

### Data mining of published literature

A literature search for the key terms “microRNA” AND “myocardial infarction” OR “acute coronary syndrome” was performed using Pubmed search engine. Full-text articles published till date (performed on 1st March, 2022) for miRs as biomarkers or prognostic markers in MI/AMI/ACS were collected.

### miR target gene prediction and functional enrichment analysis

miRNet2.0 (12) and miRWalk (13) were used to predict target genes for the differentially expressed miRs. The miR-mRNA interactions and protein-protein interactions of the target genes (PPI) were visualized using STRING interactive network visualization databases (14). The predicted targets were further assessed for their functions in biological process, molecular function, cellular component and KEGG pathways using STRING and Cytoscape (15).

### Disease-specific analysis for target genes based on text mining data

A disease-specific analysis was done using the Open targets platform (16). The target genes specific for AMI were selected after filtering for targets with text mining data available and an overall association score cut-off value > 0.2. The final list of target genes was compared with the predicted target genes of miRs from GSE148153 (7) and GSE24591 (10) for further analysis.

### Biomarker evaluation

The pipeline of outlier miR analysis (POMA) (17), a network-based bioinformatics model was used to identify candidate miR biomarkers for the diagnosis of MI/AMI. In this approach, 3 parameters namely, number of single-line regulations (NSR), transcription factor gene percentage (TFP), and unique regulated transcription factor genes (UTP), were measured for identifying the candidate biomarker.

## Results

### GSE24591 (10) dataset analysis

**FIGURE 1:**
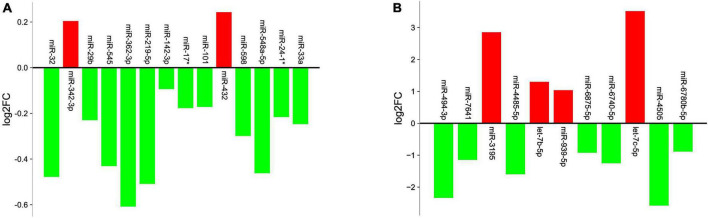
Graph illustrating the differential expression levels of miRs obtained from **(A)** GSE24591 dataset **(B)** GSE148153 dataset. X-axis represents the miRs and Y-axis represents logarithmic fold change (log_2_FC) of the miR expression levels. Red columns indicate upregulation; green columns indicate downregulation.

Analysis revealed 284 differentially expressed miRs, among which 42 miRs were statistically significant (*p* < 0.05). They were further filtered using multiple testing adjusted *p*-value less than 0.01, which resulted in 14 miRNAs (Table 2). 12 miRs were down regulated and 2 miRs were up regulated, as shown in Figure 1A. Target gene prediction revealed 4,129 targets consisting 2,907 unique targets and 1,222 shared interactions.

**TABLE 2:** Differentially expressed miRs (*p* < 0.01) obtained from the datasets expressed as log fold change (log_2_FC) and –log10 *p*-value.

Dataset	miR ID	log_2_FC	−log_10_ *P*-value
GSE24591 (10)	miR-32	–0.478	5.028
	miR-342-3p	0.204	4.811
	miR-29b	–0.230	4.742
	miR-545	–0.430	4.706
	miR-362-3p	–0.608	4.130
	miR-219-5p	–0.509	3.999
	miR-142-3p	–0.094	4.329
	miR-17*	–0.177	3.816
	miR-101	–0.172	3.649
	miR-432	0.242	3.472
	miR-598	–0.298	3.436
	miR-548a-5p	–0.462	3.411
	miR-24-1*	–0.215	3.309
	miR-33a	–0.246	3.220
GSE148153 (7)	miR-494-3p	–2.338	6.939
	miR-7641	–1.143	4.951
	miR-3195	2.851	4.237
	miR-4485-5p	–1.596	3.564
	let-7b-5p	1.297	3.836
	miR-939-5p	1.036	3.376
	miR-6875-5p	–0.919	3.474
	miR-6740-5p	–1.250	3.162
	let-7c-5p	3.515	2.883
	miR-4505	–2.575	2.764
	miR-6780b-5p	–0.885	2.757

*Passenger strand miRs.

### GSE148153 (7) dataset analysis

The analysis between controls vs. cases revealed 89 differentially expressed miRs, which after adjusting for *p*-value cut-off < 0.01, resulted in 11 miRs (Table 2). Among these, 7 miRs (miR-4485-5p, miR-494-3p, miR-6740-5p, miR-6875-5p, and miR-7641) were down regulated and 4 miRs (let-7b-5p, miR-3195, and miR-939-5p) were up regulated in the case samples when compared to controls, as represented in Figure 1B. Target gene prediction for the 11 miRs revealed 10,009 target genes consisting of 3,541 unique targets and 6,468 shared gene interactions.

### Combined analysis of GSE148153 and GSE24591 datasets

The combined analysis revealed that there were no miRs overlapping among the 2 datasets, however, shared target gene interactions were observed. The predicted target genes of all the miRNAs were further assessed by POMA analysis which revealed that 3 out of 20 miRs had UTP = 0, meanwhile let-7b-5p had the highest UTP value (UTP = 313). Table 3 elucidates the total number of target genes predicted for each miR along with unique targets and number of transcription factor genes uniquely targeted by the miRs. The complete list of predicted target genes and the unique transcription factor genes are given in Supplementary Tables 1, 2, respectively.

**TABLE 3:** Distribution of predicted target genes, unique targets and UTP value for the miRNAs differentially expressed in GSE148153 (7) and GSE24591 (10) datasets.

miR ID	Total targets	Unique targets	UTP
miR-32	379	267	56
miR-342-3p	861	663	120
miR-29b	32	27	4
miR-362-3p	477	372	66
miR-142-3p	872	673	111
miR-101	256	192	61
miR-548a-5p	30	23	7
miR-24-1*	1	1	0
miR-33a	1	0	0
miR-494-3p	1,180	525	65
miR-7641	137	65	6
miR-3195	23	13	5
miR-4485-5p	186	89	13
let-7b-5p	4,898	2,281	313
miR-939-5p	835	238	37
miR-6875-5p	110	41	0
miR-6740-5p	55	19	3
let-7c-5p	2,172	90	15
miR-4505	181	76	3
miR-6780b-5p	232	105	14

*Passenger strand miRs.

### Protein-protein interactions and gene enrichment analysis

**FIGURE 2:**
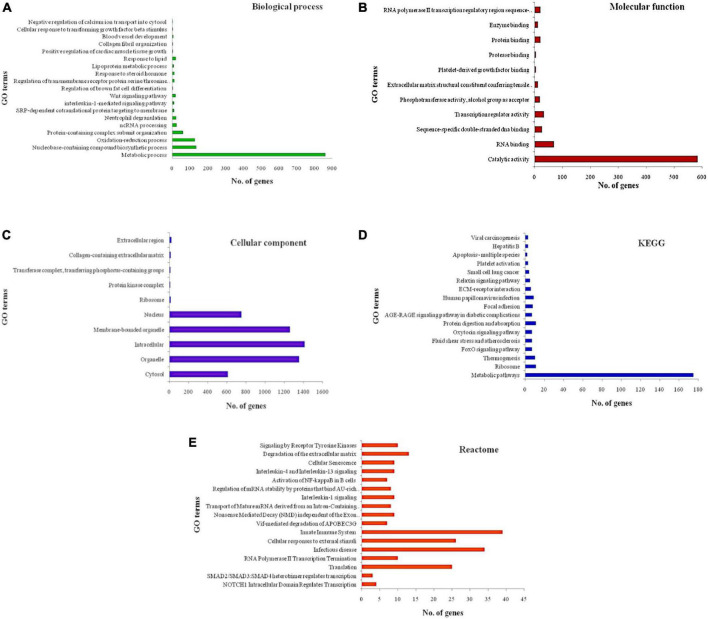
Significantly enriched gene ontology terms for the predicted targets **(A)** biological process **(B)** molecular function **(C)** cellular component **(D)** KEGG pathways **(E)** reactome pathways. X-axis represents the number of genes associated with each gene ontology (GO) term and Y-axis represents GO terms. Longest bar indicates the highest number of genes associated with the GO term and shortest bar indicates the least number of genes associated with the GO term.

STRING database revealed that of the 25 miRs obtained from both the datasets, only 8 miRs (let-7b-5p, let-7c-5p, miR-494-3p, miR-101-3p, miR-142-3p, miR-29b-3p, miR-32, and miR-342-3p) possessed target genes with significantly enriched functions. The graphical representation of the significantly enriched gene ontology terms for the predicted targets is shown in Figure 2. A representative image of the PPI interaction network for the predicted target genes and miRNA binding sites of the targets are shown in Supplementary Figures 1, 2.

Some of the most significantly enriched terms included positive regulation of cardiac muscle tissue growth, lipoprotein metabolic process, regulation of brown cell differentiation, oxidation-reduction process, advanced glycation end products and its receptor (AGE-RAGE) signaling pathway, relaxin signaling pathway, extracellular membrane (ECM)-receptor interaction, signaling by receptor tyrosine kinases, interleukins (IL) −1, −4, and −13 signaling and activation of nuclear factor kappa B (NFKB) in B-cells. Further exploration using Cytoscape revealed several relevant pathways for the predicted targets including BRCA1 associated RING domain 1 (BARD1) signaling events, Notch signaling pathway, nuclear receptors in lipid metabolism and toxicity, role of calcineurin-dependent nuclear factor of activated T cells (NFAT) signaling in lymphocytes, receptor activator of nuclear factor kappa-B ligand (RANKL/RANK) signaling pathway, fibrosis, protease-activated receptor-1 (PAR-1) mediated thrombin signaling, adipogenesis, cardiac hypertrophic response, glypican-3 network, calcium regulation in cardiac cells, myometrial relaxation and contraction pathways, fibrin complement receptor-3 signaling pathway, brain-derived neurotrophic factor (BDNF) signaling pathway, angiotensin II receptor type 1 pathway, Phosphoinositide 3-kinase (PI3K)/AKT/mammalian target of rapamycin (mTOR) signaling pathway, RhoA signaling pathway phosphoinositides metabolism, and renin-angiotensin-aldosterone system (RAAS).

### Disease-specific target gene analysis

The open targets platform revealed 1570 target genes specific for AMI, which on further filtering resulted in 12 genes, namely—HMG-CoA reductase (HMGCR), coagulation factor II (F2), prostaglandin G/H synthase 1 (PTGS1), peroxisome proliferator activated receptor gamma (PPARG), peroxisome proliferator-activated receptor alpha (PPARA), beta-3 adrenergic receptor (ADRB3), angiotensin II receptor type 1 (AGTR1), interleukin 1 receptor type 1 (IL1R1), cycling dependent kinase inhibitor 2B (CDKN2B), membrane metallo endopeptidase (MME), adenosine A2B receptor (ADORA2B), and cyclin-dependent kinase inhibitor 2A (CDKN2A) that were overlapping with the predicted targets for the miRs from both the datasets used in this investigation. Among these, 9 genes were targeted by let-7b-5p and 3 genes (PPARG, CDKN2A, and PPARA) were transcription factors uniquely targeted by let-7b-5p and miR-342-3p.

### Bioinformatics analysis of validated miRs from data mining

The literature survey provided 40 studies that have reported miRs as biomarkers for diagnosis and prognosis of AMI/ACS. About 22 miRs have been reported to possess biomarker properties for both diagnosis and prognosis (18–53), as shown in Supplementary Table 3. Some of these include miR-1, miR-21, miR-499, miR-30a, and miR-133b. Target prediction of the candidate miRs validated from data mining (published literature), revealed 13,233 targets including genes like NK2 Homeobox 5 (NKX2-5), aldehyde dehydrogenase 6 family member A1 (ALDH6A1), E2F Transcription Factor 5 (E2F5), and Forkhead Box D1 (FOXD1). The targets were further filtered by POMA analysis which revealed that all miRNAs, except miR-133a, possessed UTP > 0, and the highest UTP value of 107 was observed for miR-34a. Gene enrichment analysis of the predicted target genes revealed top 100 significantly enriched terms including muscle organ development, transmembrane receptor protein tyrosine kinase signaling pathway, NF-kappaB binding, low-density lipoprotein particle binding, cytosol, membrane-bounded vesicle and PI3K/AKT activation. Supplementary Figure 3 illustrates the summary of the *in silico* work flow along with findings obtained in this study.

## Discussion

In the present study 2 datasets (GSE148153 and GSE24591) (7, 10) were analyzed which consisted of differentially expressed miRs in AMI patients compared to controls. Analysis using GEO2R database revealed 11 miRs for GSE148153 and 14 miRNAs for GSE24591 dataset but none of them were found to be overlapping among the 2 datasets. This could be due to difference in the source of sample in each of the datasets. The miR profiling was performed in platelets obtained from participants in the GSE24591 dataset; while the GSE148153 dataset used serum samples. Data mining for candidate miR using previously published literature also revealed 22 miRs which, except let-7b, did not overlap with those observed among the studied datasets. Combined analysis of the 2 datasets revealed that let-7c-5p was the highest up regulated miR with a fold change of 11.43, whereas miR-4505 was the most down regulated with a fold change of 0.17. The overall target genes predicted for the miRs in both datasets was 14,138 which were found to be involved in various essential processes and pathways including fibrosis, leptin signaling, adipogenesis, cardiac hypertrophic response, AGE-RAGE pathway, PI3K/AKT/mTOR signaling, and RAAS pathways. Disease-specific analysis of the target genes using the open targets platform tool revealed major target genes in AMI such as HMGCR, PPARG, PPARA, AGTR1, IL1R1, and CDKN2A, which were uniquely targeted by let-7b-5p and miR-342-3p. Moreover, let-7b-5p was found to have the highest UTP based on POMA analysis.

let-7b-5p was significantly up regulated (fold change ∼2.5) in AMI patients in GSE148153 dataset. On the contrary, the expression of let-7b-5p was reported as significantly down regulated in AMI patients (*n* = 18) compared to controls (*n* = 30) and exhibited a moderate predictability for AMI diagnosis with AUC value ranging between 0.85 and 0.89 (21). Similarly, miR-342-3p was up regulated in AMI patients, according to the GSE24591 dataset. However, a study by Wang et al. (6) reported a significant down regulation of the miRs in exosomes derived from the plasma samples of 12 convalescent AMI patients. This discrepancy could be due to different sample types used for evaluating the expression levels and the time point used for sample collection. Evidence suggests that exosomal miR-342-3p reduced myocardial injury *via* SRY-box transcription factor 6 (SOX6)-mediated apoptosis and transcription factor EB (TFEB)-mediated autophagy. In addition, *in vitro* analysis revealed that the dysregulation of miR-324-3p impaired its cardioprotective ability by targeting SOX6 and TFEB (54). The expression levels of most of the miRs detected in GSE148153 and GSE24591 datasets have not been evaluated in AMI patients in the past. Studies for evaluating the expression of these miRs (especially let-7c-5p, let-7b-5p, miR-342-3p, and miR-4505) in larger prospective cohorts are required for evaluation and validation of predictability of these miR biomarkers especially in relation to the AMI/MI events in high risk patients or occurrence of secondary events post AMI/MI. We were able to prioritize the candidates for prospective evaluation in AMI patients of south Indian origin using the publicly available datasets and also published literature *via* this investigation.

GSE148153 dataset analysis was performed on available data from four males and one female patients with MI. The data was re-analyzed after removing the female sample, to assess any discrepancies in the miR expressions. The sub-analysis revealed 7 differentially expressed miRs (*p* < 0.01) namely, miR-494-3p, miR-7641, miR-3195, let-7b, miR-4485, miR-21, and miR-6088. Except miR-21 and miR-6088, all the miRs were significant in the initial analysis (before removing the female sample). However, a reverse expression pattern was observed in this sub-analysis, whereby those miRs upregulated in the initial analysis (miR-3195, let-7b) showed downregulation after removing female sample. Similarly, the downregulated miRs in the initial analysis (miR-494-3p, miR-7641, miR-4485) were upregulated after removing female sample. In the initial analysis, miR-21 and miR-6088 were not statistically significant. Supplementary Table 4 describes the differentially expressed miRs in the sub-analysis as compared to those obtained in the initial analysis.

Several genes were predicted as direct targets of the differentially expressed miRs from the datasets and text mining data including forkhead box O1 (FOXO1), NFKB1 and RANKL. FOXO1 is a transcriptional factor which plays vital roles in the regulation of cell metabolism in various tissues, particularly in the heart and endothelium. It plays a major role in glucose and lipid metabolic pathways, atherosclerosis and cell survival. A recent study (55) evaluated the expression levels of FOXO1 gene in 138 non-ST elevated MI (NSTEMI) patients with and without type 2 diabetes, which revealed that FOXO1 was elevated in NSTEMI patients irrespective of the presence of diabetes. In addition, the levels did not decrease even after treatment. FOXO1 has also been associated with diabetic cardiomyopathy. RANK and its ligand RANKL are predominantly involved in bone metabolism and immune system. RANKL has been reported in several bone related diseases, especially osteoporosis and bone metastasis (56). RANKL is a cytokine which binds to its receptors (RANK) on the surface of osteoclasts to promote the maturation of macrophages and is especially responsible for bone remodeling. On the other hand, it has also been observed that soluble RANKL baseline levels could predict the risk of adverse cardiovascular events including myocardial infarction. Moreover, this binding of RANKL to RANK triggers the activation of NFKB signaling cascade which can have either protective or deleterious effects. RANKL is reported to influence myocardial inflammation in case of cardiac overload and has also been associated with adverse cardiac remodeling events post-MI. Moreover, following myocardial infarction, the RANK/RANKL/osteoprotegerin (OPG) axis gets activated (57).

The predicted target genes of the differentially expressed miRs among the datasets analyzed were found to be involved primarily in leptin and insulin signaling, PI3K/AKT/mTOR, AGE-RAGE, and RAAS signaling pathways. Leptin is essential for maintaining body weight and energy balance. A deficiency of leptin or defects in the components of this signaling pathway can lead to obesity, one of the major risk factors of AMI (58). Leptin phosphorylates PI3K which in turn activates AKT and mTOR (59). The PI3K/AKT is one of the major pathways involved in controlling the survival and functions of cardiac muscle cells by regulating downstream molecules, especially mTOR. In addition, PI3K/AKT/mTOR pathway primarily regulates protein synthesis in cardiomyocytes (60). Ning et al. (61) investigated the role, functions and mechanisms of miR-494 in ischemia/reperfusion-induced cardiomyocyte apoptosis and autophagy. The miR-494 expression was down regulated in hypoxia/reoxygenation-treated H9c2 rat myocardial cells when compared to control cells and silent information regulator 1 (SIRT1) was identified as its target gene. Moreover, a knockdown of SIRT1 elevated the phosphorylation levels of PI3K, AKT and mTOR in the treated H9c2 cells, indicating that miR-494 had protective effects against hypoxia/reoxygenation-induced cardiomyocyte apoptosis and autophagy by targeting SIRT1 (61).

RAGE is expressed in a various cells and tissues of the body including heart, kidney, lung and skeletal muscles. It has been reported in the pathogenesis of several diseases, especially aging and cardiovascular diseases. AGE-RAGE signaling had been shown to play an important role in the progression of atherosclerosis *via* oxidative stress and pro-inflammatory response (62). It is essential for the generation of reactive oxygen species, autophagy, apoptosis, inflammation, endothelial permeability and dysfunction. Till date, there are no studies that have investigated the involvement of AGE-RAGE pathway in AMI. Due to its diverse and pivotal roles in the cardiovascular system, a detailed understanding on the AGE-RAGE signaling transduction in the pathogenesis and progression of AMI is essential. Another significantly enriched pathway for the predicted target genes is the RAAS pathway, which is comprised of renin, angiotensin II and aldosterone. It mainly functions in regulating the blood volume and systemic vascular resistance (63). This indicates the importance of miRs in modulating RAAS signaling pathway. RAAS blockade with angiotensin converting enzyme (ACE) inhibitors or angiotensin receptor blockers (ARBs) has been reported as beneficial for disease outcome in AMI patients *via* preventing/reversing endothelial dysfunction and atherosclerosis (64). RAAS had been frequently modulated by several drugs such as enalapril and losartan for the management of heart failure, AMI and hypertension (63).

Furthermore, the expression pattern of the miRs and their respective target genes were analyzed using the GSE148153, GSE24591, GSE60993 (65), and GSE48060 (66) datasets. The target gene expression profile obtained from GSE60993 and GSE48060 datasets did not reveal any statistically significant difference among AMI patients and control group. The relationship between the miRs and their target gene expression (gene expression data obtained from GSE24591 dataset) in the above mentioned signaling pathways is given in Supplementary Table 5. Nevertheless, having evidence of target gene expression in relation to the miRNA changes within the same cohort prospectively shall provide clear evidence for the prognostic value of the miR expression in AMI. In addition, the target genes were further looked up for the association of known variants in the genes with different phenotypes related to cardiovascular disease, including BMI, hypertension, lipids, dyslipidemia and pulse rate using the knowledge portal for cardiovascular diseases (67). Majority of single nucleotide variants (SNVs) in protein kinase C alpha (PRKCA), ATPase plasma membrane Ca2 + transporting 1 (ATP2B1), phosphoinositide-3-kinase regulatory subunit 3 (PIK3R3), protein patched homolog 1 (PTCH1), and interleukin 6 (IL6) genes were significantly associated with lipids, especially total cholesterol and LDL-C. The variant in ATP2B1 gene (rs2681492) was associated with CAD and rs2810915 variant in PTCH1 gene was significantly associated with atrial fibrillation. Supplementary Table 6 describes the various SNVs in the target genes that are associated with phenotypes in CVD.

Our future perspectives include determining the association of the enriched pathways in AMI by performing detailed systematic review. In addition, a custom-array of these miRs as biomarkers would be developed for evaluating the association of the candidate miRs expression with secondary events in AMI patients (UNIAMI study) using plasma samples as the input source.

One major limitation of the study is that we could identify only 2 datasets that was relevant to our analysis criteria. Moreover, systematic literature search has not been incorporated in this investigation. Due to a lesser number of samples from available datasets, there is a greater chance of missing out vital miRs that play a contributory role in the pathogenesis of MI.

## Conclusion

In conclusion, this *in silico* study has prioritized miR candidates, predicted their targets, and their functional enrichment to establish the molecular mechanisms and role in the pathogenesis and progression of AMI. The analysis revealed the regulation of significantly expressed miRs like let-7b-5p, let-7c-5p, miR-4505, and miR-342-3p in important functions/pathways including PI3K/AKT/mTOR, AGE-RAGE, and RAAS by directly targeting AGTR1, FOXO1, NFKB1, RANKL, HMGCR, and AT-1. Functional studies on these identified signaling pathways are required to understand and prove the role and mechanism of action of these miRs and target genes.

## Data availability statement

Publicly available datasets were analyzed in this study. This data can be found here: The gene expression omnibus (GEO) repository, accession numbers: GSE148153 (https://www.ncbi.nlm.nih.gov/geo/query/acc.cgi?acc=GSE148153), GSE24591 (https://www.ncbi.nlm.nih.gov/geo/query/acc.cgi?acc=GSE245 91), GSE60993 (https://www.ncbi.nlm.nih.gov/geo/query/acc.cgi?acc=GSE60993), and GSE48060 (https://www.ncbi.nlm.nih.gov/geo/query/acc.cgi?acc=GSE48060).

## Ethics statement

Ethical review and approval was not required for the study on human participants in accordance with the local legislation and institutional requirements. Written informed consent for participation was not required for this study in accordance with the national legislation and the institutional requirements.

## Author contributions

PV and CRSU designed and conceived the study. PV contributed to the literature review, data collection, bioinformatics analysis, and wrote the manuscript draft. KB, MG, and SK reviewed the manuscript and provided inputs for data interpretation. CRSU contributed to the final approval of the manuscript and agreed to be accountable for all aspects of the work. All authors contributed to the article and approved the submitted version.

## Supplementary material

Supplementary Figure 1 A representative image of the PPI interaction network for the predicted target genes. Colored nodes (circles) indicate direct/first line interactions among the genes. Purple and blue color lines denote known interactions determined experimentally and from curated databases, respectively. Black, dark green and light green lines indicate co-expression, neighborhood gene and interactions derived from text mining, respectively. Thickness of the lines indicates strength of data support.

Supplementary Figure 2 MicroRNA binding sites on target genes for prioritized miR candidates obtained using TargetScan Human 7.2 database.

Supplementary Figure 3 Flow chart illustrating the summary protocol and findings of *in silico* analysis using bioinformatics tools. “*” Denotes 25 miRs obtained from GSE148153 and GSE24591 datasets only; “#” denotes partial list of the vital functions/pathways involved in myocardial infarction.
