# Supplementary material for: Prioritization of microRNA biomarkers for a prospective evaluation in a cohort of myocardial infarction patients based on their mechanistic role using public datasets
Source: Front Cardiovasc Med. 2022 Nov 3;9:981335. doi: 10.3389/fcvm.2022.981335 (PMC9668885; doi:10.3389/fcvm.2022.981335)
Supplement: Supplementary file 1 [file Data_Sheet_1.docx]

| **miR**  **Table S1: Target genes predicted for the miRs obtained from GSE148153 and GSE24591 datasets using miRWalk and miRNet 2.0** | **Predicted target genes** |
| --- | --- |
| **let-7b-5p** | ACPP,ACVR1,AGL,JAG1,AKT2,AMPH,SLC25A4,ASPA,ATOX1,ALDH7A1,ATP6V0A1,AUP1,BCL7A,BFSP1,BGLAP,BMP7,VPS51,CA12,CAPG,CBFB,ENTPD6,CD81,CD151,CHD3,COX7B,CSNK1D,CTBP2,CYP1A2,CYP2J2,DRG2,E2F2,ENG,EP300,ETFA,F2,FANCD2,ACSL1,FEN1,FLII,GAPDH,GPI,GPM6B,GPX7,GTF2I,GYG1,GYS1,GYS2,HIST1H1C,HARS,HRAS,AGFG2,HES1,HSF2,HSPA1B,IFNB1,IGHMBP2,RBPJ,IMPDH1,IMPDH2,ITGB5,RPSA,LPL,MAB21L1,MARS,CD99,MIPEP,MOV10,MPG,ABCC1,MTRR,MYO1C,NDUFA10,NEDD4,NFATC1,NFATC3,NFKBIA,CNOT2,NRDC,NVL,PA2G4,PAFAH1B3,PAK1,PCBP2,PCCB,PCYT1A,PDGFRA,PDK1,PFKM,PGM1,PLCB3,PLK1,SEPT4,POLD2,POLR2H,PPM1G,PPP1R7,PRKD1,MAP2K2,PSMD9,PTGFRN,PVR,PCYT2,QARS,RALB,RPL18,RRBP1,RXRB,SALL2,SC5D,SEMG2,SMARCA1,SMARCA4,SUMO2,SNRPE,SP100,SPN,SPR,TROVE2,SSR1,ST13,TCOF1,NR2E1,TPBG,TPD52L2,TST,TYMS,UBE2I,UGT8,ZNF3,ZNF148,ZMYM2,SLC30A1,BRPF1,ARHGEF5,HIST1H3B,DHX16,CUL3,DENR,TNFSF12,TRIM24,CCNK,EIF2B3,CCNA1,WASF1,UBA3,UBE2M,MAP7,PAPSS1,USP10,HGS,DDX21,ADGRG1,COPS2,FADS2,PCYT1B,BAG5,SOX13,CLCA2,HS2ST1,PUM1,USP15,HNRNPDL,NUBP2,FARP1,MARCH6,AKR1A1,WARS2,TUBA1B,SCML2,NDRG1,CD2BP2,ZER1,SEC23B,UNC13B,IPO8,RPP38,POLR3G,RAI1,KIF1C,AHCYL1,WDR4,TUBGCP2,PPARGC1A,TXNL4A,POP1,STIP1,GCN1,SLC27A2,TMEM115,ATE1,ABCB8,DDX20,PMF1,DNAJC8,SCAF8,CPEB3,RPIA,PDCD11,RBM34,RRP1B,VWA8,PPRC1,ARHGAP26,EPB41L3,TTLL12,ATG4B,SYNE2,PDS5A,ADGRL2,DNMBP,ZCCHC11,RRP8,ABCB10,SEC11A,CARHSP1,MKRN2,ARFIP2,PLXNB2,TMEM2,RAB38,IFIT5,CIZ1,NIPBL,POLR1A,AHCTF1,PTPN23,VIRMA,MMACHC,PTCD1,IPCEF1,DNM3,APPL1,TES,TIMM9,NFU1,TOX3,MAT2B,OSTM1,COMMD9,UHRF1,CPSF1,SLC25A24,NOP53,EHD4,TRNT1,LACTB2,VPS28,HGH1,DDX41,SFMBT1,MTFP1,FAM49B,ERGIC3,TAF9B,MRPS33,CHMP3,CHRAC1,TERF2IP,MIOS,MIER2,DDX49,ARL15,RBFOX1,PPP1R12C,NSMCE4A,WDR55,GPATCH4,PIGG,NSUN2,DNAAF5,DUSP23,C1orf27,INTS11,PIH1D1,CCDC186,GPATCH1,ARHGAP17,ANKZF1,SDAD1,KLHL11,BBS7,STEAP3,QRSL1,WDR33,LSG1,TMEM63B,SUPT20H,UBE2Q1,TRMT1,LRRC40,YEATS2,IARS2,MAP7D1,POLR3B,SLF2,CNDP2,RIOK2,DDX28,UTP6,DBNDD2,NXT2,KYAT3,C1GALT1,PITHD1,RALGAPB,SPRYD7,NHSL1,BIRC6,KIDINS220,XPO5,ZNF687,NCOA5,SLC25A19,BCORL1,GALNT11,ERAP2,DNAJC1,NXN,ZNF106,GZF1,CPEB1,ANAPC1,TBC1D15,ELOVL1,CCDC71,NT5DC2,MRPS24,NOL6,WNK1,PYCR3,NABP2,KXD1,ASPSCR1,FTO,WDR25,IPO4,LIN28A,GEMIN7,ISOC2,NAA40,FAM57A,CCDC134,MRM1,CPED1,DOCK5,NAA25,FOXRED2,ZNF606,NAA15,WDCP,TRABD,AARSD1,CPTP,INTS5,SLC25A32,SLC38A1,GDPD5,RCC1L,C6orf62,MED25,EMC6,ATAD3B,CDCA7,WDR75,ANTXR1,POLR1B,FAM96A,FAM213A,MIEN1,CCDC115,LZIC,GTPBP3,IL17RC,PLXDC2,MPND,REPS1,EAF1,PIP4P1,ADCK2,DHX57,L3MBTL4,YTHDC1,C11orf52,OXNAD1,NAF1,MGME1,SLC38A5,MFSD3,TOE1,MBD6,OSBPL10,CTHRC1,DCD,RFFL,GABPB2,PIGU,NUP35,CMC1,DAB2IP,FAM84B,USP54,CENPV,UNC13D,HIPK1,SLC35F1,AFG1L,TTC9C,GATC,ZNF841,NBPF15,RNASE10,SPATA12,PSMG4,NOMO3,ANXA8,ANXA8L1,C1orf112,CFH,GCLC,SEMA3F,HOXA11,MEOX1,FAM214B,PRKAR2B,CROT,PDK2,ABCC8,SPATA20,USH1C,ARHGAP44,MARK4,RPUSD1,TEAD3,NADK,CYTH3,AASS,IL32,SEC62,CSDE1,FAM76A,POMT2,STARD3NL,CD9,BTK,LYPLA2,ANLN,TMEM159,PRICKLE3,ALOX5,CALCOCO1,MAP4K5,CLDN11,MVP,UFL1,RGPD5,ISL1,ATP2C1,WWTR1,CYP24A1,CD74,NCDN,RUNX3,SPAST,AQR,FHL1,DERA,GCLM,CCDC28A,PHF20,TYMP,VIM,ARNTL2,NUP160,BAK1,FUT8,TIMP2,AGA,ZFYVE16,BEST2,RAB27B,PSMA4,PARP3,TG,DCUN1D1,CNTLN,ANO2,WWC3,HDAC9,RRM2B,ZNF800,ELN,DKK3,LIMA1,PIK3CB,MPHOSPH9,FNIP2,FSTL4,PLEKHH1,USP13,ATP11B,TARBP1,STYK1,SNRNP40,ZNF112,EPN1,AHRR,HAGH,DLX3,SPA17,ABCA7,SBNO2,HMG20B,AP3D1,ZNF76,DGKA,ANKRD44,ADAT1,ME1,THUMPD1,KIF26A,KLF6,PRKCZ,PDK3,HYAL2,HDAC4,IFI35,INPP5A,REEP1,PSME4,IFT80,MAST4,PIGB,OSBPL3,HLTF,FAM50A,RDH11,PRKACA,LIMS2,LNX1,CHFR,CRMP1,MRVI1,PANX2,CLCN4,DHRS9,PICALM,MRPS34,PPP2R2C,TEAD2,MGLL,NUAK1,ANO8,ZNF37A,ATP12A,DLG1,SEC31B,SART3,PLXNA2,ANKRD13A,ACACB,XAB2,CTTNBP2,TOP2B,NFKB2,PAK3,FAM76B,MAP2,MCCC1,LAMP3,FAP,N4BP2,PCM1,SLC1A3,LXN,TNS1,RAPGEF3,RIMS1,SMARCA2,COL5A3,DLGAP4,CADPS2,PHLPP1,ATP8B1,STRADB,KCNK2,RNF13,BCKDHB,P2RX5,DIS3,CHMP2B,APOB,KIF3C,ATRX,WDFY1,ABCB1,CTTN,NME8,SNX10,TMED2,NOX4,ACOX3,BAX,TMPRSS11E,MT3,NID2,SF3B2,COQ9,ZNF343,RBBP9,TMEM230,OAS1,PXN,FXYD5,ZNF302,CDIP1,SPTLC1,PCBP4,YPEL3,ICAM1,STRN4,SI,GNPTG,NAT14,OSBPL8,TXNL1,ORC6,RGS17,TGM1,PHGDH,CLSPN,TGFB2,PTGS1,TBC1D2,SEMA4G,ZNF184,IFT74,JAK2,ABLIM1,ERMP1,RAB18,PRTFDC1,MYO9B,IZUMO4,DERL3,CRYBB3,HIRA,HSCB,TOMM22,DNAL4,CHKB,BIK,TSPO,PNPLA3,SGSM3,COCH,VCPKMT,PLEK2,GSTZ1,TMED8,PAPLN,PSMB5,ACIN1,GMPR2,PNN,PCIF1,NDRG3,PSMA7,SLCO4A1,TCFL5,ARFGAP1,EEF1A2,C20orf27,TRIB3,RSPO4,PLCB4,MYL9,KIF3B,OXT,CST3,MAP1LC3A,MYL12A,CEP192,LAMA1,ANKRD12,POLI,STAG2,ABCD1,FMR1,RP2,JADE3,HTATSF1,VGLL1,TIMP1,KLHL4,RBM3,TAF7L,KLF5,UGGT2,RGCC,INTS6,MSLN,PHKB,CENPT,ZNF423,CCDC113,SLC7A6,TSC2,FOXF1,METRN,HERC1,BMF,ZDHHC2,EYA1,DECR1,GDAP1,EMC2,INTS10,TUSC3,KCTD9,KCNN4,RELB,RNASEH2A,DKKL1,STX10,SNAPC2,IL27RA,TNNT1,RASAL3,SYDE1,TBCB,APLP1,MRPL4,ETFB,TYK2,RABAC1,CYTH2,TMEM205,BCAT2,PPP2R1A,LSR,HPN,ETHE1,AVL9,SP4,MPP6,HOXA5,BRAT1,IQCE,HOXA13,TAX1BP1,SNX8,PTPRZ1,MOSPD3,SERPINE1,POU6F2,RARRES2,BLVRA,PRKAG2,YKT6,SLC1A1,PRUNE2,PTGR1,AK1,CDC37L1,PLGRKT,KANK1,CREB3,DDX58,GLIS3,RAPGEF1,SH3GL2,PHYH,LIPA,TNKS2,DKK1,SMC3,DNAJC12,CRYBA1,KPNB1,CCDC47,ALDH3A1,C17orf75,CYTH1,CCL2,KAT2A,CNTNAP1,PTGES3L-AARSD1,ABCC3,LUC7L3,DUSP3,SLC16A6,MMD,DHRS7B,RCVRN,SLAIN2,DCUN1D4,CHIC2,NMU,INPP4B,CLCN3,SOD3,GLRB,SNX25,NCAPG,CRYAB,CTSC,ELP4,VWA5A,CCDC86,RNF141,MLEC,PTGES3,WNT5B,RAD51AP1,CREBL2,ARHGDIB,GTF2H3,FZD10,MDM1,ENO2,C2CD5,COL12A1,BTN3A3,TDP2,NEDD9,TULP1,KCTD20,CD83,RBM24,PRDM13,ALDH5A1,SOBP,FIG4,SLC39A7,PPP2R5D,MRPL2,EXOC2,NUDT12,MAN2A1,MRPS30,HMGCR,PDE8B,CDH6,NPR3,FAM172A,GNPDA1,LIFR,TXNDC15,DPYSL3,DBN1,NPHP3,CEP70,RBP1,SLC25A36,FXR1,CBLB,GNB4,PODXL2,PLSCR4,CISH,DNAH1,TNNC1,ADAM23,TP53I3,TANC1,SLC30A3,MPV17,INO80B,LOXL3,DOK1,EVA1A,FANCL,IL1R1,IL18R1,PROC,PRKD3,DLX2,KYNU,C2orf42,TIA1,EPAS1,ALMS1,MARK1,PAPPA2,RNF19B,ARHGEF2,NCF2,PLA2G4A,RGS2,SRSF11,PLEKHM2,LGALS8,SIPA1L2,RPF1,GBP3,PRPF3,IPO13,PIK3R3,PRRC2C,VAMP4,CD3EAP,STAG1,KLF7,USP35,SLC16A7,CASQ2,ABCG2,RARRES1,MFSD1,PPL,ELL2,GDA,TJP2,PTBP3,WDR34,GLE1,NR4A3,KDSR,ACYP1,LTBP2,MLH3,RBM25,ALDH6A1,COQ6,ATAD2B,YPEL5,CNRIP1,EPCAM,IFIT2,GPAM,PANK3,TEK,RCL1,MOB3B,CD274,NUP43,CXorf21,ACAT2,PDZD11,SMAD9,KBTBD7,ALG5,UTP20,ARL1,GLT8D2,SORBS3,TNFRSF8,CRISPLD1,SCPEP1,MND1,TAS2R10,PYROXD1,ZNF211,GJA8,DESI2,POLR3GL,PIK3CA,TMEM156,TMEM54,CLCC1,POLK,FLT3,UBL3,SASH3,ZC3H7A,ODF2L,RWDD3,CCDC18,HOXA7,GLIPR2,RECK,TAF1L,EGR2,CCDC91,PKN1,OPTN,ATF1,SPATS2,ORMDL2,HOXC13,HOXC11,HOXC12,TNFAIP6,ACVR1C,G0S2,PLA2G12A,FAM210B,PI3,WFDC3,PLCG1,CHST8,STAMBP,ZNF45,OARD1,SLC35B3,EREG,BBS2,MT2A,DOK4,EFNB2,RNF113A,BMP4,PTGER2,NT5C,SLC2A4RG,PSD4,CD70,TRIP10,FOSB,VASP,TMX4,BMP2,PCID2,CAPNS1,RBM42,PRDX5,IRF3,RRAS,ASL,ELK1,ZBTB1,NXF5,ZC4H2,ZNF484,ABHD8,TSPAN8,PTPRB,TAS2R5,SYNGR3,WDR24,GNGT1,STYXL1,YWHAH,CDC42EP1,TPST2,RAC2,EMC4,MKLN1,FLNC,MYO1B,EIF2AK4,VPS13C,LOXL1,EGLN3,ART1,CDKN1C,ERMARD,MOSPD2,CNN1,APOE,GADD45G,FAM98C,ATP8B3,BMP15,CRB3,OLFM1,UBAC1,COL5A1,TMEM160,CLIP1,PLXNA3,HABP4,BPIFA2,C1QL1,ZNF428,TEX101,CDX4,MRPS25,SH3BP5,TBC1D5,NAPSA,GFPT2,ACAP3,C1orf159,NINJ1,IL13RA1,TNS4,TOP2A,PODNL1,PPARG,HSD17B7,PTPRE,TMEM128,PNISR,TRIM47,VPS13B,DAP3,NASP,KANK4,DCTN4,ZMYM5,LGR6,DCLK1,TRPC4,RFXAP,BEX2,ZNF414,MACROD1,C12orf29,SBF2,GSC,IER3IP1,EDEM1,DPH6,TSPAN2,VTCN1,NGF,IL2RA,SOX5,RERG,LRP4,HOOK1,PRPF38A,DSC2,DSC3,TPGS2,ERCC5,ETS1,ANXA1,FAM189A2,PSAT1,NT5E,AKIRIN2,CGA,PRRG4,FAM186B,TSPAN31,PAN2,AHI1,HEY2,PKIB,CPM,CEP350,CHRND,USP37,STAB2,PLXNC1,MED4,NUDT15,GPNMB,ACTL6A,ODF2,SLC31A2,POLR1E,DNAJB5,GRHPR,HINT2,ARHGEF39,PPIL1,GMPR,SLC22A7,KIAA0319,GCM1,MDC1,CLPS,FGFBP1,FHDC1,RAB30,PI15,GGH,TGS1,NEK1,DDX60,TRIM29,SLC37A4,C11orf1,ALKBH8,ARHGAP29,IFI44,SLC44A5,FBXO11,ATRAID,CEP55,PLCE1,DBR1,AOX1,PPIG,CCDC54,MNS1,FAM13A,HERC5,HNRNPD,PRKG2,CCNG2,PPA2,ERP27,KIF21A,AEBP2,TMEM117,VAMP1,SCAF11,INHBE,PHLDA1,LGR5,LUM,NEDD1,TDG,SDSL,ESYT1,VPS37B,RHOF,CUL4A,FRMD6,SYT16,JDP2,FBLN5,SLC25A47,NIPA2,SLC12A6,HDC,GTF2A2,TPM1,ARRDC4,BBS4,TGFB1I1,ADAMTS18,MYOCD,CTRL,RPRD1A,C18orf21,CBLN2,DPP9,IFITM3,LMTK3,ADAMTS10,ZNF473,FAM71E1,EPHA2,SH3BGRL3,PLK4,BCL10,TINAGL1,TMEM61,MRPL24,XPR1,LHX9,ECM1,CGN,ARNT,VASH2,ADAM15,RAB13,SLC27A3,SLC39A1,RIT1,TTC13,LYST,SYT2,MALL,FBLN7,UBXN4,UBR3,PHOSPHO2,NYAP2,CPNE9,RBMS3,IL17RD,NXPE3,ABHD10,AGTR1,TRPC1,TCTA,MANF,EIF2B5,CENPC,EPHA5,SNCA,CISD2,PDGFC,MYO10,IQGAP2,GIN1,BDP1,TSLP,GLRA1,TENM2,TNFRSF21,PPP1R18,LGSN,RARS2,RSPO3,SHPRH,VIP,RBAK,IGFBP3,TMEM47,SLC16A2,NONO,ZNF711,ZNF185,ATP6V1B2,GNRH1,ERLIN2,TACC1,SYBU,FAM83A,SLC39A4,UHRF2,CDKN2B,CDKN2A,C9orf72,AUH,LCN2,DPH7,HERC4,ADIRF,GLUD1,ANKRD1,INA,DGKZ,NPAT,B3GAT3,SOGA1,YTHDF1,TRPT1,TM7SF2,MKX,CTF1,KLHL1,LYPD1,LYPD6B,ADRA2A,GPM6A,PRSS23,DOCK1,IL18,SEC24D,PLBD2,TEX30,ME3,PTPRO,EPS8,ACAD8,EDNRA,NR3C2,PIGF,RNF144A,ASAP2,FLI1,CACUL1,BEND6,MZT2B,PSTPIP2,SPC25,PDE3B,ZFP36L2,IGSF10,DSPP,MBNL1,CAPSL,NADK2,MARVELD2,STK32B,CWC27,MR1,DAB2,SCOC,CLGN,CETN3,RASSF3,PTPRR,LURAP1L,FBXO36,CEBPG,IMPACT,ANKH,CDH12,ANGPT1,PITPNC1,C4orf19,UCHL1,MIA3,TNIK,SH3RF1,PDLIM3,SORBS2,C21orf91,SLFN13,EME1,HSPA13,ZCCHC10,LRGUK,PDIA4,KCTD18,FAM126B,FZD7,DEPTOR,FMN2,SLC26A2,RASA2,CLIC2,ADAMTS3,DPY19L4,TDRD9,MTERF3,CD109,UNC5D,TBC1D31,PRR14,TIMP4,SSBP3,TMED6,DHRS1,AASDH,MX1,DGKI,BRAF,AP3S2,GRHL3,UBXN11,HPD,CDC25C,EIF5B,SPATA2,COPG2,FGF17,CDA,PINK1,FBXW5,SV2A,CSRP1,CBR3,PTMS,PSMB4,HK2,NAE1,ATP6V0D1,ZYX,ABR,CCDC28B,ANKLE1,CCDC58,G6PD,AGPAT3,VAV2,SLC2A6,C9orf116,COX6B2,DEDD2,FDPS,FAM189B,CCR5,UBQLN4,LY6E,FBXL13,MFSD12,ITGA5,JOSD2,FAM171A2,RAVER1,SYCE2,ZG16B,ASRGL1,RPS6KA4,ZYG11B,ZSWIM5,PEF1,SYNC,TSSK3,ALPL,CCDC27,DIRAS3,NEXN,GBP2,ATXN7L2,KCNT2,PEX19,SLAMF6,PKDCC,CCDC138,EN1,NOSTRIN,S100A11,PGLYRP4,PAQR3,ANTXR2,CLDN1,SLC22A15,ELF3,SSR2,RNF25,NEK10,AZI2,STT3B,IFI16,EIF5A2,CDS1,PTPN13,THOC7,ATXN7,TIPARP,SMIM14,APBB2,PCOLCE2,CXCL1,SNRK,RFC4,PIGX,S100P,ERMAP,SGMS2,MST1R,HMGB2,FBXO8,FAM160A1,ITGA2,ELOVL7,NDUFS4,SERINC5,EBF1,TLX3,CREBRF,STXBP5,RAET1E,TRA2A,STEAP1,CTSB,TNFRSF11B,PDP1,RPP25L,SNAPC3,NUDT2,ABCA1,MAMDC2,PRSS37,ALDH1A1,RASEF,ZHX1,MID1IP1,STOML2,ZCCHC24,DNAAF2,NEMF,CDX2,COMTD1,ARHGEF40,C10orf82,TTC7B,SLC39A13,TC2N,IFI27,PTER,ABTB2,HTRA1,CEP57,JAM3,ADAMTS15,SPATA19,GPT2,PCBD1,CYB5R2,WDR72,CDYL2,C16orf46,CASC4,AGBL1,SLFN5,ANPEP,STAT6,SCG5,TSC22D4,CCNDBP1,SMAD3,EVA1C,PHB,TTC16,SLC27A4,PRRX2,ISLR2,HDHD2,MYO5B,OR51I1,RAB8A,TUBA1A,NFKBID,SPINT2,C19orf33,PSCA,GLOD4,RILP,WDR81,CYB5D2,ANGPTL4,TMEM88,KRT24,TMEM99,GHDC,ZNF598,MLST8,RAB3IL1,SAC3D1,FAM83B,DDIT4,UBTD2,PCMTD1,IRF2,DEGS2,TAP1,BDKRB2,MTCL1,CDKN2AIP,SNRNP48,ZNF30,LRP1B,PKIG,GSTM4,ABHD15,C2orf68,PLA2G4F,SLC35G2,PPIC,HSPBAP1,FAM110B,MN1,NSMCE1,CCDC126,RAB3B,TBC1D10B,GPRIN1,HSPB3,SLC33A1,PLEKHA2,GPR183,CLIC3,ACTRT2,LDB2,RAC3,GSX1,ROBO1,TPST1,TM4SF1,ZNF768,SIMC1,FAM161A,CDK1,B3GNT2,CST2,ADORA2B,NUDCD2,IRF2BP1,ZNF296,SDR16C5,CHCHD7,HTRA3,KBTBD2,NDUFA3,NUDT6,TANC2,ZNF160,PDGFD,PLAC1,S1PR1,C11orf24,MFN1,JAGN1,C9orf16,RBKS,GRIK1,SMR3B,CHST11,KRT9,PDE7B,ZNF524,DSEL,ETFDH,LPAR3,ZNF274,RHNO1,CFAP46,MLLT3,FRMD5,TLN2,TVP23B,SRGAP2C,SCG2,EPHX4,LRRC15,KRCC1,FRMD3,TEFM,ISG20,GNG12,MANEA,RASGRP1,NAA16,HOXC5,RPL38,ZNF621,EGFL7,NBEA,MYEOV,MRGPRF,BNC2,COQ2,COMMD1,GLRX,GPR137,SNCG,DAB1,PPP1R14B,VEGFB,CSPG4,SCAI,LRFN4,C11orf80,TOMM20,TIGD3,NET1,GPR160,GOLIM4,RBM4,XXYLT1,TLR6,FAM174A,GLIS1,CHRNA9,C11orf45,PELI3,IL20RB,BRMS1,THAP6,SEZ6L2,CA5A,TEX36,ATR,UBE2C,VCPIP1,PDIK1L,PSMD2,PPM1E,CSRP2,DDIT3,CLTB,PCSK1,MCTP1,POLD4,DRAP1,C11orf68,TOMM5,PRIMA1,ETV4,SWI5,A2M,DOK7,TUBB6,NUPR1,SSNA1,CSTF3,TCP11L1,SPHK1,ANAPC2,PRR15,FOXC2,BOK,NFATC2IP,FAM89B,SEC24C,FBXO46,TALDO1,CASKIN2,ZBTB38,MAGEF1,NAP1L5,ACOT4,IRX3,CD163L1,ZNF518A,TMEM187,CCDC184,ZBTB41,MLF1,C2orf69,BOLA1,KCNG2,SLC25A20,CPNE7,C5orf46,HYI,DAND5,GPC5,HTR1D,APOBEC3B,B3GNT3,C3orf80,PRKRA,TIGD2,ZNF571,NRIP1,MB21D2,PCGF5,SLC36A4,HOXC10,OXTR,CMTR2,GPR137C,BBS12,TRAPPC5,F2R,TMEM102,SETD2,MEX3D,TMEM259,ENPP7,LDOC1,SYNM,AP1S2,FBXL6,C1S,GLTPD2,CLN8,CACNB4,TRAPPC6B,SATB1,BRICD5,IGIP,CRIP2,PLCXD3,GLUD2,ZNF721,CEP63,MTA1,MCEMP1,NKX2-5,FHL3,PMCH,RIPK4,MX2,ALYREF,NOG,TRIM52,EMILIN3,TMPRSS2,ADAP2,CRELD2,PPP1R2,TSSC4,MAP7D2,KNTC1,SOCS3,PDE4B,FAM110C,NDUFA12,OSBP2,PRR16,F8,SEMA4B,RPS27L,MANEAL,SIGIRR,IFITM2,TNFAIP2,MAGEA11,C12orf56,SMYD3,KRT6B,STAC3,ROR1,MUC1,NR2F2,AHNAK2,SHC4,WDR53,THNSL1,ATP6V0C,LEMD1,TAL2,CD300LF,PIP5K1C,CYP4F12,BLOC1S4,TMEM212,KRT3,PCLO,CYP4F3,UBE2H,C17orf58,CYP27C1,ZNF397,KRT16,KRT14,TMEM17,MITF,SPATA21,RNF220,ZNF385C,PERM1,PEAR1,ZFP69B,DMBT1,COL14A1,S100A3,GPR89B,ZC3H6,AGAP4,PP7080,IGFL1,JAKMIP3,NDOR1,CLN3,DPYD,IDO2,SLC4A5,GJB3,FAM111B,SP6,KRT77,PNRC2,KAZN,SERPINA3,S100A4,HRCT1,FAM217B,ASB13,ARMCX4,TCEAL3,TPK1,XRCC2,MYO6,ZNF136,TECPR2,HSH2D,ZNF418,DAPK1,COL27A1,S100A2,ZNF700,MAML3,ADA,ANKRD36B,PDLIM7,SLC39A10,AP2A1,ANXA4,WDR5B,WDR45,KIAA1671,ACSL5,ZNF785,CYSRT1,SLC22A4,ZNF165,PELI1,UAP1L1,SLC22A5,ADARB1,IPP,TTC30A,COL4A6,TOPORS,MFAP5,SPTAN1,CFD,KIF13B,TEAD4,ELOVL2,CCDC151,AKR1B10,HMGN5,ZNF251,SZT2,CSF2RA,STYX,TMEM116,HYLS1,ZNF813,GFPT1,TMA16,TTC37,MAGEA1,MSRB1,SMURF1,CDC42BPB,PLXNB3,FAM169A,CNOT7,ZNF521,ARHGAP11A,TOX,RUSC2,FICD,LTN1,CCDC152,TOP1,L1CAM,DZIP3,GPRASP1,ZFP2,MFAP3L,NAGA,TGM2,PJA2,INF2,EFCAB2,FANK1,FOXO6,MAFB,ZDBF2,BMPR2,AGPAT1,MRPL38,NEU1,CARD16,LY6G5C,PRR3,PPP1R11,MZT1,LRRC10B,TRIM13,C4orf46,TMEM170B,ATP6AP1L,CCDC85C,DNAJC19,SERPINB5,GPX3,RNF208,DENND1B,CRIP1,TRIM59,MLLT11,MXD3,ARHGAP19,KRTAP4-12,LBH,PPP1CB,DDAH2,IRF9,GALT,SPINK13,IFRD2,ZBED1,SMTNL1,LRRC69,UBE2QL1,MYL5,TAF9BP2,TNFRSF25,TSTD1,ZNF579,C6orf226,FANCG,TIGD1,SLC12A8,FAM185A,HSBP1L1,SRRM5,ERVMER34-1,LTA,OST4,DHFR,HLA-DPA1,ZNF879,ZSCAN31,KIAA0040,PNMA6A,FAM200B,RNF103,RBM14,AQP1,RPL36A,ARPC4,ARPC1A,PSG2,PSG4,STON1,NFS1,IFITM10,ETV5,HBB,N4BP2L2,NAIP,TMEM158,ZNF718,FPGT,ZNF432,HP,ZBED6,TUBB3,POC1B-GALNT4,THTPA,BLID,PMF1-BGLAP,XKR7,CCPG1,EPPK1,SLC6A14,ZNF587B,LIX1L,DOC2B,DCP1A,AK6,ARHGAP23,TUBGCP5,ORAI1,PCGF2,ZNF670,GGNBP2 |
| **let-7c-5p** | CCNC,DLX4,DNMT1,EWSR1,FOXO3,GARS,HOXD1,HSPA4,IL10,JARID2,LDHA,LECT2,SMAD6,MPL,MYCL,RARB,RPL3,RPS4Y1,RPS13,RYR2,SLC12A2,TAF5,TAZ,TTF1,UBA52,ITGA10,COPS3,NUMB,EIF3A,MBTPS1,RNF7,SLK,NUP58,SNUPN,OLFM4,METAP2,NUDT21,NRM,R3HDM2,SNW1,KIF1B,CSTF2T,DNAJC16,SIRT1,GORASP2,TRIB2,SBDS,RAPGEFL1,PCF11,CNNM2,LAMTOR1,FANCI,ETNK2,HHAT,PSMG2,WDR61,FAM103A1,RAB34,METTL25,DCTN5,RHOV,RFX6,CEP170B,HES5,PPAN-P2RY11,MBTPS2,CTNNA1,CLNS1A,SENP1,HMOX2,PSMA2,ATL2,RAB11FIP5,CASP6,TMEM19,TADA1,KCTD15,CNNM4,GPR85,AATK,PRKDC |
| **miR-3195** | NPBWR1,MSX2,PHLDA2,VDR,VGF,ARC,CASZ1,VWA7,MIB2,SIX5,C2orf82 |
| **miR-4485-5p** | CACNA1A,MPZ,PTAFR,RAB27A,KDM5A,TTC4,VHL,RND2,TAF1B,PIAS2,SLC28A2,GLYAT,TAB1,SYNRG,KLF8,SEPHS1,MESD,AIPL1,ABHD12,CCDC69,GPR82,HCAR1,CRCP,ANGPTL3,MCTS1,FAM216A,STRN3,PEX5L,TOLLIP,ZSCAN2,OGFOD1,PARVA,METTL2B,ASAH2,KCMF1,GATAD1,IFT22,GPBP1,ZNF655,ZNF419,ZBTB3,ADM2,SYNPO2L,CEP76,RSPH3,CNDP1,HIST1H2AH,ATCAY,ANGEL2,ZNF682,C1orf158,WDR31,WDR92,SLC43A2,TMIGD2,TCEANC2,ICA1L,SGO1,NIPAL1,PGBD4,ZNF384,FAM153B,ZFP82,LINC00632,MMAB,NLRP10,CAVIN4,CDNF,AGAP9,RNF222 |
| **miR-4505** | ACVRL1,ADAR,SLC25A6,ARL2,CD4,CSK,GJA5,GRM1,GUK1,MSH5,PDE6B,PRKCA,PEX2,RPL37A,SLIT1,STAC,STX4,ADAM17,TAF1,MLX,AKAP1,HIST2H2AA3,NEURL1,DLG5,FGF19,PARP2,PLIN3,DMRT2,FGFR1OP,CDC37,MAPK8IP3,FBXW8,HECA,TMEM248,KLHDC8A,RNF126,TMEM63C,XPNPEP3,POPDC2,CENPO,L2HGDH,PTDSS2,PARD6B,WDR73,R3HDM4,SYTL3,LEAP2,METTL6,TMEM184A,C10orf67,CEP57L1,ZNF284,SEPT14,ZNF321P,PCP4L1,HIST2H2AA4 |
| **miR-494-3p** | ARHGAP5,ARNTL,ATF3,BMI1,CFTR,CNR1,SLC26A3,ETF1,GCH1,IL1RAP,MCC,MYH2,NCL,NHS,PPP1CC,MAP2K1,PTPN12,RAP1B,SDC1,SLC8A1,SSFA2,TBCA,UGT2B17,ZBTB25,EVI5,CDS2,BASP1,KIF2C,FOXJ3,LATS2,TMOD3,HSD17B12,WNT16,TM6SF1,MINDY2,TMLHE,TRIM36,AJAP1,WDR45B,RRAGD,ZBED2,FYCO1,EFCAB1,MAP1LC3B,GALP,CCDC149,ARHGAP12,INO80C,DCAF4L2,C2CD4A,SAMD15,TMEM64,SPATA13,EYS,ZNF736,TMEM178B,DPM1,FUCA2,LAP3,CFLAR,HCCS,RALA,MYLIP,PSMB1,SYPL1,SS18L2,PLAUR,UBR7,CLK1,CCDC88C,ZRANB1,BCLAF1,RNF19A,TLL1,TDP1,GOPC,MRPS10,CLPTM1L,HEXB,JKAMP,MCUR1,LETMD1,THOC3,PLEKHA5,NOP58,MRPL43,SLC2A3,RNF4,OAT,PDIA5,FTSJ1,LAPTM4A,TRIP13,MCM2,EED,BCS1L,SRI,SPAG5,CAPZB,ITCH,XRCC5,STK17B,CPNE3,NFX1,DIMT1,EPDR1,ZFAND6,MMP2,DNM1L,ANKRD10,TGDS,RPLP0,ERP29,NUDC,SEL1L3,ZC3HC1,SCFD1,G2E3,GEMIN2,DPYSL2,PSMD5,TMED1,GADD45B,EIF3L,KDELR3,POLDIP3,RBX1,RBM23,EIF5,PSMC1,SRP54,TM9SF1,SEC23A,MMP9,PFDN4,NOP56,ADNP2,PSMD10,PRPS2,DNAJC3,CEMIP,TMEM87A,EIF3J,EIF3E,NFKBIB,ASF1B,VRK3,PDCD5,PIAS4,LIG1,WDR83OS,TMEM147,DNAJC2,TFPI2,DNAJB6,LFNG,CASP2,PDAP1,PHF14,EXOSC3,MAP3K8,RAD51C,TRIM37,RPS6KB1,CBX1,TMEM97,OCIAD1,ELF2,NEIL3,CCDC34,CHORDC1,C11orf58,VPS29,NOP2,CDCA3,CHPT1,C12orf57,SRSF9,PAK1IP1,HINT3,COX7A2,HARS2,TTC1,GOLPH3,CSNK1A1,NIT2,SNX4,SPCS1,EEF1B2,RPS15,RTN4,EFHD1,MOB4,KDM3A,ABCB6,RAB3GAP1,DHCR24,CACYBP,MAD2L2,BCAS2,GNPAT,RSRP1,OSBPL9,TNFAIP3,SGK1,RNF146,CCNI,CTNNAL1,PPP6C,NDUFA8,HSDL2,VPS4B,EIF2B2,GNA13,NUDCD1,KIF18A,KHDRBS1,LRIF1,KIAA1191,ARL4A,SMU1,ACO1,PLAU,ZWINT,PRDX4,KBTBD4,SDC4,SRSF6,RNF114,VAMP7,MPHOSPH10,RRP36,MED20,SNRPB,HNRNPR,EIF2S2,MCF2L,PRMT1,TRAP1,CTAG2,COX7C,AAMP,GNG11,CASD1,TWSG1,PALLD,TXNDC17,SNX9,ARPC1B,ATXN10,CEP85,SH3BGRL,PPT1,RPL27,THOC6,RAF1,NUP210,EFR3A,TBC1D14,SEC61G,NIP7,POLR3F,POMP,SWAP70,MEIS2,PSRC1,SPIRE1,TMEM106C,IL6ST,FST,RBM17,DDB2,ACP2,GNL2,TMED7,NREP,WDR36,ISCA1,SNX14,ORC3,KCNK1,URB2,COG2,PWP1,COG3,SMPD4,ABI1,NIPSNAP3A,DSCC1,DERL1,CNPY3,RABGGTB,CYP1B1,RAB1A,LRPPRC,STAMBPL1,KIF20B,SSB,NAB1,USP8,GLCE,AP1AR,FGF5,G3BP2,SCARB2,HADH,SEC24B,NDUFA9,CERS5,C12orf10,RAB20,SLC38A6,UBE2Q2,IQGAP1,PARN,UQCRC2,NPEPPS,FN3KRP,PSMB6,KIAA0319L,LMO4,ALDH9A1,MGST3,TOR1AIP1,PIP5K1A,DTL,SNAPIN,MEIS1,DUSP11,AFF3,RAB5A,SLC25A38,FIP1L1,LYAR,USP53,RPL37,BTF3,ZMAT2,TMEM181,CHMP7,RPL7,MED22,ARFGAP2,INTS4,CHEK1,UBC,DCP1B,NDUFC2,AKR1C2,SAV1,NBAS,SCHIP1,TMEM123,LPCAT1,RMND5A,GTF2E1,ENAH,TSEN2,ANKRD40,TMEM237,MED7,NTAN1,CDC42SE2,MIS18A,CBR1,U2AF1,RUSC1,CPSF4,LARP4,SENP3,RBBP4,FUBP1,ARPC5,PEA15,CAPN2,RPP14,RYK,TMEM41A,RPN1,SAP30,ABCE1,NDUFAF2,GPX8,SLU7,OSGIN2,COX6C,FZD6,NFIL3,ZNF22,PRPF18,PACSIN3,MOAP1,TMED3,BLCAP,DOLPP1,MGAT2,TMEM208,ZBTB43,APEX2,SLC50A1,MRPL1,BUB1,UGP2,TMEM42,UBE2E1,COMMD5,EXOSC1,CAMTA1,RPS7,PRNP,ZNF131,AFF1,DAG1,PSMD1,STAT5B,DPY19L1,RPL15,BANF1,TMEM70,RMI2,RUFY1,ZDHHC13,SLC38A9,ARIH2,NDUFAF3,FBXO34,TMEM107,C14orf119,CUEDC1,SIAH2,CYB5D1,ADI1,SKA2,RPL35A,NIPSNAP1,NR2C2AP,SS18L1,SNN,MORF4L1,LYPD6,MT1X,TCEA1,NHEJ1,FAM166A,NDUFA4,CD55,EPHB4,XRCC6,MYL6B,FAM72A,VKORC1L1,CXorf40B,MRPL21,FAM114A1,MCMBP,NOL8,ZNF248,WDHD1,RPL10A,ZNF830,GK,RPL39,GIGYF2,CNEP1R1,LIN52,DIO2,SACM1L,SLC48A1,ZNF254,CPNE1,TOMM6,PPP3R1,NOL7,RPS28,ZBED5,ADSL,MIF,ARFGAP3,GNG10,EID1,MTRNR2L8,MTRNR2L1,RPL36A-HNRNPH2 |
| **miR-6740-5p** | ARF1,IARS,WNT10B,DYNLL1,DYRK4,MALT1,NMNAT2,ANKS1A,DCAF12,P3H2,ZMAT1,RPTN,CCDC39 |
| **miR-6780b-5p** | ALDH3B1,ARRB2,STS,CACNB1,DDX11,DUSP8,DUSP9,EMX1,GPR20,GPR26,GPR35,HOXD3,HOXD13,JUND,LIMK1,NAB2,OLR1,FXYD1,PYCR1,RREB1,SLC9A1,VPS72,TLR5,UGT2B4,WNT9B,RAB36,TESMIN,SUSD6,RGP1,SMYD5,ABHD2,ERG28,NXPH3,RBPJL,COPZ1,WDTC1,ANGEL1,PADI1,BICRA,TMEM69,ARL6IP4,AZIN1,EPB41L4B,WBP1L,DCAF16,VPS37C,ALPK3,UBE2Z,ASXL3,FAM49A,ITM2C,GLIS2,ADO,SELENOI,SCIN,MARCH9,ZFYVE27,HSPB6,MPLKIP,ZBTB46,ZNF467,CRTC2,ZSCAN25,SDK1,TMEM86B,DYNAP,CCDC137,ANKRD45,SNX19,IQSEC3 |
| **miR-6875-5p** | AQP2,VEGFA,GABRQ,TRAPPC9,LMF2,TGFBR3L |
| **miR-101-3p** | ACVR2B,BIRC5,APP,RHOA,ATM,ATP5B,NKX3-2,CCND1,BCL9,PRDM1,CAPN2,RUNX1,CCNF,CD81,CDH5,CDK8,CDKN1A,KLF6,COX10,DCTD,DSC1,DUSP1,ELAVL2,ELAVL3,EXTL3,EYA1,EZH2,FBN2,FOS,MTOR,GCLC,GNB1,GRSF1,HFE,MNX1,HNRNPAB,HNRNPF,HNRNPU,DNAJA1,HSP90AA1,ITGA3,STMN1,LBR,LMNB1,MBNL1,CD46,MAP3K4,MET,MITF,COX2,MYCN,MYO9A,NACA,NAP1L1,NOTCH1,SLC11A2,PCCB,PDK1,PIK3C2B,PIK3CB,PIM1,PIP4K2A,PPP1CC,PPP2R2A,PRKAA1,PRKAB1,MAP2K1,PTGS2,QDPR,RAB5A,RAP1B,RORA,SHMT1,SLC7A2,SMARCD1,SMN2,SPAG1,HSPA13,TAF13,ZEB1,TFAP4,THRB,TNFAIP1,HSP90B1,UBE2A,UBE2B,VEGFC,ZNF124,ZNF207,ZNF223,REEP5,ARID1A,CDC7,EEA1,CBX4,STX16,EED,CDC123,SGPL1,INA,ATG12,GPR50,CTR9,TTC37,N4BP1,BZW1,MLEC,DAZAP2,TSC22D2,ZC3H11A,PPP4R1,RANBP9,MBNL2,TRIB1,IPO7,STAMBP,TGOLN2,LEFTY1,SUB1,UGT2A1,KIF2C,SNRNP27,SNRNP35,DIDO1,NUPL2,KLF12,ICK,SACM1L,KDM6B,DICER1,PHF3,SUZ12,TSPAN12,CDC42EP4,SNHG1,MOB4,LTN1,ANKRD17,TOR1AIP1,GMEB2,BLOC1S6,DIMT1,TNRC18P2,ANKRD11,USP25,CERS2,ZBTB21,GOLGA7,GLRX5,NT5C3A,IER5,ZBTB7A,PEX5L,NLK,KDM3B,SIX4,PANK1,IL20RB,SLC38A2,RNF111,TET2,MBTD1,MFSD6,CMTM6,DNAJC28,LRRC1,MRGBP,PSPC1,ZNF654,40787,RIOK2,NXT2,KCNQ5,C8orf4,SPIRE1,RARS2,TBX20,RTN4,ZNF490,USP36,KIAA1456,JCAD,LRCH2,RNF213,RAP2C,ABHD17C,ZNF350,TGIF2,FAM217B,PRDM16,CPEB1,CERK,SLC30A5,MRPL44,WNK1,MPPE1,KCTD14,RNF219,L2HGDH,C10orf88,RMI1,FBXO11,RAB11FIP1,RAB33B,FAM103A1,SLC25A33,LZIC,ATG4D,MTSS1L,LYSMD3,FOXP4,RAB39B,ARAP2,AEBP2,NAA30,AP1S3,DCBLD2,CARNMT1,NEK7,ZNF480,C1orf52,B3GALNT2,KLHL23,FAM84B,ZNF645,ZDHHC15,DENND5B,PGBD4,ZNF567,ZNF100,ZNF800,KCNG3,ZNF431,AGO4,TVP23C,TMEM192,CCDC125,MMS22L,CHAMP1,SREK1IP1,RPL7L1,PABPC1L2A,DCAF12L2,ATXN1L,NACA2,ZNF284,LANCL3,ANKDD1A,NANOGNB,FAM69A,BEND4,LIN28B,RBM12B,C1orf147,UGT2A2,HSPE1-MOB4 |
| **miR-142-3p** | LPP,PPP1R2,SKP2,TMEM59,U2SURP,FBXO3,CNIH4,LYRM4,MTMR9,FYCO1,EDEM3,ZNF682,TMED4,ACACA,AGTR2,ALAD,APC,ARF4,ARNTL,ATP2B1,AZF1,C2,C4BPB,CCNG1,CDC25C,CDKN1B,CNN3,COPA,CRH,CSNK1G3,CSRP2,DAG1,EGR2,STX2,EVI2B,LRRC32,GFI1,GNAS,GNB2,GOLGA1,HMGB1,HMGA1,HOXA7,HOXA10,HSD17B4,HSPA1B,CYR61,IL1A,IL6,IRAK1,IRF1,KIF5A,LAMC1,LOX,SH2D1A,MARCKS,KMT2A,TRIM37,NAP1L2,NEFH,OSBP,SERPINA4,PRKCA,PSMB5,PSMD11,PSMD12,RAB2A,RGL2,RPE,RPS19,S100A11,VPS52,SCD,TROVE2,SYPL1,SYT4,THBS4,TSPAN6,NR2C1,TSPYL1,UGT2B17,XRCC1,YES1,YWHAE,ZNF217,DDX39B,CUL5,FZD7,HIST1H4D,RGS5,PPFIA1,LGR5,PROM1,RTL8C,CHRNA6,CLDN12,HGS,SOCS6,ROCK2,SCAMP1,MORF4L2,MTFR1,SOCS5,PUM1,USP6NL,IP6K1,GOLGA5,PARP2,DNAJB6,ABCC9,SLC25A13,STX6,CALCOCO2,TFG,SEC23A,DDX17,SDCCAG3,MALT1,MAN1A2,MORF4L1,DNAJB4,SLC7A9,KAT7,FICD,POLI,CA5B,MGLL,ATF5,COPG1,INPP5F,ELL2,CLUAP1,GPD1L,ZCCHC14,DIP2A,PSME4,SIK2,MMD,ZMYND8,TMEM245,MTCH1,CBY1,TPSG1,ZNF473,INTS7,PTPN23,FAM98A,KBTBD2,GLCE,RTL8A,IRF2BP1,SLC17A5,RAB30,FLVCR1,EEF2K,RRM2B,TAS2R7,TMED7,GOLT1B,HSD17B12,DCTN4,GINS2,ARL15,TBC1D13,RAB39A,FAM208B,SLC35F6,CDCA4,AKIRIN2,CEP192,ETNK2,PCMTD2,STRBP,LRRC59,YOD1,DOCK10,DEPDC1,PEX26,BTBD7,FAM222B,ENAH,ZNF701,NSFL1C,PCDHGA10,PCDHGA2,PCDHB14,RNF20,C11orf16,AKR1B10,TWSG1,PDSS2,SCYL3,KIAA1191,ZNF608,MTUS1,TAOK1,DOCK6,USP37,ZNF529,WIZ,MANBAL,ACBD3,TSEN34,SECISBP2,ATG9A,RIPOR1,PANK3,AGBL2,TUBAL3,TNIP3,SLC35E1,C16orf70,PPP1R14C,RNF170,NBPF3,AKT1S1,TMEM101,MSANTD4,TNRC18,SPPL2A,TBRG1,TMEM209,KRTAP4-5,PPIL4,SIGLEC10,MCFD2,BOD1,ACBD5,TIFA,DTD1,MMGT1,ARHGAP12,ADPRHL1,MBD6,KCTD12,PSTK,C11orf74,ARL14EP,SPPL3,WHAMM,MPLKIP,C7orf31,EIF5AL1,MESP2,C18orf25,ARL6IP6,FREM1,HSFY2,ZFPM1,ZNF676,FAM209A,TET3,C9orf72,TMEM136,RICTOR,NUDT8,SGMS1,MRGPRX1,ARL10,DPY19L4,CLEC4D,ZNF678,VSIG1,MROH7,SRRD,LINC00598,PCDH18,RGPD5,CLEC19A,DENND2D,DAPK1,MAPK10,CABP4,TMEM252,SKP1,PTPMT1,KY,CCDC9B,HEY2,RIF1,PARVA,HES2,TRDMT1,CRP,FNDC9,ZNF99,ZIC4,GSG1,TRDN,DOK1,ZBTB10,DCTN3,OR4N2,SCN4B,RBM39,CEP78,CCDC163,RGS3,SNX13,ZNHIT6,RBFA,FBXO10,FBXO32,ZXDB,HMGB3,FMNL3,GUCY1A1,TUBGCP2,R3HCC1L,CEP41,CUL3,GRIA1,HARS1,KCTD1,ZNF501,TP53I11,ARHGAP45,CLPB,UEVLD,CDK15,ZNF211,GPR161,JAKMIP2,ST3GAL6,PGR,PTGS1,CALCRL,DYNC1I2,ITPRIP,ELF2,B4GALNT1,HOMER1,P2RY6,NBPF20,RHOH,LCTL,SURF4,SMC1A,NLGN4X,GAB3,ZNF107,PGRMC1,MTA3,CDV3,FAM71F1,PUM2,LRIT2,PITPNB,VOPP1,AMZ1,SLC2A14,PPIL6,KCTD20,TMEM68,PRMT2,KLHL1,SPATA13,GRID2,RALYL,STK32A,FDFT1,ZNF286A,CEP70,ZNF302,FAM104A,CECR2,ZNF717,IGF2BP2,MAPK4,PDE4B,CAMSAP2,CNGA3,ZCCHC10,SNX3,ZMIZ2,CDK19,DLG2,NHLRC4,ZNF773,PRKACA,TSHZ1,EPHB2,CPNE5,OR4E1,HNRNPK,DNMBP,ADAM11,OPCML,NUMB,WNT9B,ZFP30,RUNX3,DLG4,PDGFD,DDHD1,CSRNP2,ANKRD13C,OR2T1,SCRT1,CPVL,GABARAPL1,AIF1L,GNGT2,PCDHA5,KRTAP4-8,NLRC5,USP48,ZMYND12,ATP13A4,LLPH,TMEM107,PARD6G,NT5C1A,HDGFL2,SYAP1,JAM3,RNFT2,PWWP3A,PCBP4,NEK9,RBM18,GABRG3,SOX6,CNTNAP3,BRWD1,XKR4,PLEKHG4B,GNG2,CDKN2B,UPF3B,SLC46A1,TBX5,EDARADD,APBB3,PPARGC1B,PAQR8,MYOZ3,PLCD3,CD109,DCN,SLC12A6,TLR4,ANKRD54,BACE1,SLC39A11,MTFMT,EHD4,ZNF700,C15orf40,TBC1D20,PDCD2,SKA3,ATOH7,SENP8,MRPL10,CCSAP,SSBP3,TRAF6,ANXA11,TTC30A,NXPE1,FAM161B,SCFD2,DNAJC18,TBCEL,DOK6,SDK1,SNX33,MFSD4B,ODF2,ZNF114,FNDC5,RASGRP3,NMNAT2,CD200R1,CD8A,IL15RA,DMBX1,APBB2,ST6GAL1,TSHZ2,ZDHHC23,ZNF776,XKR6,FAM133A,ETFBKMT,SLC30A8,LRATD2,XG,PIGN,PDE12,PPM1B,DCX,TBC1D26,CERS3,IL21R,DCDC1,PKLR,C20orf203,RASSF5,CLEC10A,PRKACB,BCKDHB,RBPMS2,BCLAF3,CPM,ANKRD46,EMB,LONRF2,RSBN1L,VWC2,FAM120AOS,GABRG2,CNNM2,AMER2,GAS7,FIBIN,ACSL3,TRPM3,LTK,NAALADL2,C2CD4A,FREM2,TEX19,RAPH1,ATP6V1H,CSRNP3,MYO10,MGA,CDH8,B9D1,ZNF181,ZNF324B,THAP6,CAMSAP1,CRYL1,ECHDC1,GUK1,SPTBN2,MME,CIP2A,N4BP2,TRIM2,MYLK4,SYNJ2,FAM86B1,SPIN3,MTARC1,SMPD4,FAM71F2,SGCE,MYO3A,DUSP8,FRMPD3,CAPN13,POMT2,KCNA2,ROR1,NRG1,TMEM234,MYT1L,KAT6A,AKIP1,RAPGEF3,NAV3,GPC6,LRRC63,MIPOL1,FLRT2,CELF4,ETV3L,ABCG1,SYCP2,MLH3,UTP25,TSPAN13,OTX1,SDC3,PHACTR2,KIAA0513,KIAA0319,FCHSD2,PPM1E,ADNP2,RAB21,ZHX3,SIPA1L3,SARM1,POFUT2,ACSL6,USP22,TECPR1,C2CD2,RSL1D1,NALF2,NSDHL,KLF13,MYEF2,CYB5R1,ADAM22,SPOUT1,PHF21A,WDR1,KIF21A,GIN1,GDAP2,COMMD8,TMEM127,CLN6,PACS1,MSL2,SBNO1,RBM41,AGPAT5,PLCXD1,DCP1A,BCAP29,PCDHB7,RBM47,MRPL50,DPYSL5,BDH2,SAR1A,CMC2,BBX,ACKR3,SLC24A2,PARP11,GOPC,PIGA,NRIP3,JPH3,AICDA,KCNS2,PPM1H,LRRC47,ESYT2,GPR158,HECW2,SRGAP1,SYT13,FNIP2,NCOA5,NEUROG3,C6orf47,NECAB1,PORCN,MYH11,RANBP17,LRRC19,HAPLN4,CENPO,CHST5,NAA35,STEAP4,ZC2HC1C,DIPK2B,C1orf115,NHEJ1,MORN1,STN1,ZC3H12A,PLXNA2,CEBPG,CSN2,E2F5,FGF4,AFF2,FUT4,GPX2,HLA-DOA,HOXB5,KCNA6,KLRD1,KPNA1,MYC,NDN,NFX1,MAPK3,RAB3B,SRSF3,SSRP1,TCEA3,SEC62,VCL,STX7,SORBS2,ELP1,DENR,IRS2,SUCLA2,USP13,INPP4A,ATP6V0B,SLC28A2,CD83,BAG2,BST1,OSGIN2,MAPKAPK3,NEMF,SRSF11,MED1,CYP7B1,CD8B,PDE1C,TNFSF18,DLX6,EPHA3,ERBB4,GLI2,SMAD4,ABCA1,KIF20A,NAB1,HTR3B,SEMA3A,CORO2B,CAPZA2,NFE2,NFRKB,UQCRB,MCRS1,CHERP,ARPC1A,POLH,GLRA3,FUT9,KRT38,RAB31,RORB,ZNF81,SEC23IP,GABARAP,C4A,GRIA3,ICOS,RAB3GAP1,RRAS2,AP2A2,OR2A5,PFAS,SEC22A,N6AMT1,ZNF180,GREM1,UQCR10,PREB,HTR1F,KLHL21,DHRS7B,UBIAD1,WBP2,CNKSR2,RO60,ERV3-1-ZNF117,MINDY4B,C13orf42,C3orf85,OVCH1,ZNF738,GGA1,NOTCH2NLB,NOTCH2NLC,C4orf50,ESRP2,LOC100132202,PABIR3,NEU3,LARS2,AKT3,LOC102724488,DUS4L-BCAP29,GABRB2,C10orf67,NNMT,IKZF5,AHRR,LRIG1,CHRM2,SLITRK5,GPRIN2,RBFOX3,OR11G2,ZNF320,ALDH3B2,ANKRD18B,DENND1A,EPAS1,ERN1,ESR2,GFRA3,ACADL,CLCN5,COL4A4,KIT,PEX2,SGCD,SLC4A1,TIMP3,XDH,TPP1,IGFBP3,TGFB1,GOLGA6L1,IL17REL,C4B,GPR179,OR2L3,OR51A4,OR8B3,ZNF182,C3orf56,MXRA7,TPST2,C12orf29,PIK3R6,CENPP,CTNNBIP1,SGO1,FAM221B,NHLRC3,C2orf68,COX19,FAM131B,YTHDC1,MAP9,INSYN2A,PEG10,NUPR1,NALF1,WTIP,EVX2,FITM2,TMEM150C,TMEM218,C1orf226,IRAG1,RNF169,C2orf80,AMOT,MGAT1,TP63,FCGR3A,DCAKD,SHC1,C15orf62,AP3M2,FAM86B2,CHID1,GOLGA6L6,FAM104B,ZNF736,C3orf18,DGCR2,UHMK1,MCIDAS,TMTC1,YAP1,DHFR2,MEFV,NT5C1B-RDH14,NEK1,RPL36A-HNRNPH2,PRLR,CAV3,CCNH,PDE7A,TCF4,DPYSL2,TRPC1,PTK6 |
| **miR-29b-3p** | CRYBG1,AKT2,AMFR,AQP4,BCL2,BMP1,CALM3,CASP8,CCNA2,CDC42,COL1A1,COL2A1,COL3A1,COL4A1,COL4A2,COL4A5,COL5A1,COL5A2,COL6A3,COL7A1,COL10A1,COL15A1,DDX6,DNMT3B,DSC2,DUSP2,ELAVL1,EMP1,EPHX2,EREG,FBN1,FGA |
| **miR-32-5p** | ACAA1,ACTC1,ADAM10,ASGR2,ATOX1,ATP7A,BCAT1,BCAT2,BMPR2,DST,CAD,CCNB1,CCT6A,CD69,CDC5L,CRKL,CYP2C19,DSC3,DUSP5,EIF4EBP1,EIF4EBP2,FKBP1A,GAA,GATA6,GM2A,GOLGA4,GTF2E1,H3F3B,HIVEP1,HOXC8,FOXN2,RBPJ,IPP,ITGA6,ITGB8,LAMP2,LETM1,SMAD6,SMAD7,MAN2A1,DNAJB9,MDM2,MPP1,MYO1D,MYO5A,NEFL,NFYB,CNOT2,CNOT4,NRAS,NSF,PAWR,PAX9,CDK16,PITPNA,NPY4R,PRCP,PRPS1,PSMD5,PTEN,RAD21,RBMS2,REV3L,RNF4,RP2,RPE65,RPL9,RPL24,RPLP1,TSPAN31,MAP2K4,SLC7A1,SLC9A1,SNRPD1,SOX4,SRPRA,AURKA,ELOA,DYNLT3,TESK1,TMF1,TRIO,TSC1,UGP2,UQCRFS1,UVRAG,VDAC2,ZNF17,ZNF134,ZNF157,ZNF224,EVI5,RNF103,FXR1,SNN,GEMIN2,IFITM1,CHST1,MTMR1,CDK5R1,PER2,SYNJ1,MBD2,CCNE2,SCAF11,SLC33A1,XPR1,CYTH2,KLF4,RPL23,MED7,ITM2B,VPS4B,RAB3D,EDEM1,ZSCAN12,SERTAD2,MRPL19,SPOCK2,GIT2,TECPR2,AP5Z1,G3BP2,DENND4B,SUPT7L,JOSD1,SH2B3,ABCF2,TOB1,CNIH1,EIF1,CTDSPL,AP3S2,ZNF267,PRMT5,ARFGEF2,PAIP1,EXOC5,POLQ,HBS1L,LILRA2,TPPP,TBC1D8,ZNF277,AKAP10,DUSP10,ZHX1,B4GALT7,FKBP9,IKZF2,SBNO2,PAXIP1,KLHDC10,GOLGA8A,MAST3,KIF1B,MCF2L2,GRAMD4,CIC,UBXN4,FBXO28,KIAA0556,RPRD2,OTUD3,KLHL18,AGTPBP1,NEMP1,DOCK9,SLC39A14,RBFOX2,SGK3,DSTYK,TMEM184B,NECAP1,IBTK,SPATS2L,APPL1,EDRF1,KIF1BP,MYCBP,NARF,CNNM4,RANBP6,USP21,NPTN,TOR1B,CCDC113,C1GALT1C1,MYLIP,SNX10,PURG,BAZ2B,ASAP1,RNF141,MRPS16,KMT5B,DNAJC27,ERGIC2,GULP1,VPS54,SELENOT,GALNT7,BCL11A,C11orf24,C21orf91,MRPS21,CCSER2,XRN1,ANKIB1,PUS7,PPP1R12C,DNAJB12,ESRP1,TOR4A,UHRF1BP1,NCAPG2,MTMR10,LAX1,DUS2,ARGLU1,CCDC186,RBM28,SLC25A36,SETD5,SMU1,PLXNA3,FAM46A,G2E3,SYBU,BTBD2,PRPF40A,MFN1,ATF7IP,TEX2,FAM214A,PMEPA1,ARNTL2,MFF,OTUD7B,TULP4,ZNF695,PHTF2,CYP20A1,REXO1,GATAD2B,SLC12A5,ARID1B,CASKIN1,ZNF398,KLHL14,FAM135A,ZNF492,USP28,ZNF317,ANO8,PCTP,AGBL5,GUF1,SPCS3,MRPL17,CIDEC,PBLD,MOAP1,C17orf75,PAPD5,NUCKS1,AEN,IFT22,CASD1,UBE2Z,GRAMD2B,MIS12,GID4,ZFYVE21,C1orf35,OR2A4,VCPKMT,PPCS,TXNDC15,AGMAT,PEAK1,LONRF3,TRMT2B,PTGES2,CPTP,SLC25A32,SGPP1,FAM49A,ANP32E,C6orf62,COG3,MRO,SLC10A7,PCBD2,ZNRF3,KIAA1109,YIPF4,DNAJC30,DDI2,SLX4,PARD6B,ZNF594,GLYR1,CARD6,PRRC2B,FUT10,ZNF607,USP45,SLC45A3,RHPN2,ZIC5,OTULIN,TMEM41A,ZNF598,ARRDC4,ZC3HAV1L,UBE2Q2,TMEM44,FNIP1,TIRAP,PCMTD1,FCHO2,TNFRSF13C,NUS1,FAM129A,ZNF354B,PIK3AP1,PDZD8,FOPNL,EARS2,WDR81,IRGQ,SHE,TATDN3,LIN54,NOL4L,SAMD8,SESN3,ZNF417,ZNF98,ENTHD1,DAB2IP,SRFBP1,FAM91A1,ZNF383,DENND2C,MIER3,FUT11,ZNF721,AAED1,DAND5,TXLNA,APOBEC3F,FLCN,CCDC171,VMA21,C2orf69,MED19,MCOLN2,SPRYD4,GOLGA8IP,ZADH2,PPP1R37,NLRP9,RAB7B,ZDHHC21,XKR7,NUP43,PTAR1,NUGGC,VHLL,MUC21,ZNF772,PGAM4,ARGFX,GOLGA8J,TMEM239,C15orf38-AP3S2,MYZAP |
| **miR-342-3p** | ACTG1,BMP7,DUSP6,FOSL2,NAPG,CBX1,FAM107A,XPOT,FOXJ3,TP53INP2,KIF13A,SYNPO2L,ZNF548,PUS10,ZNF610,MMAB,ABL1,ACOX1,ACVR1B,ATF4,CALR,CD3D,NCAN,CXADR,DARS,DIAPH1,EEF1A2,EEF2,EPB41,EPHB3,F9,FPR1,GART,GJA1,GOT2,GRID1,HARS,HNRNPC,HOXB3,IDH3B,IDS,IGFBP5,IREB2,LSAMP,MLLT6,P2RX3,PA2G4,PHKG2,PKP1,PLA2G4A,PRKCE,PRKCSH,PTGIS,PTPN14,PTPRN2,PWP2,RAD23B,RFX3,RGS4,RPL15,RPL26,RPL27A,RPL37A,RPS3A,RPS9,ATXN7,SELPLG,SLC6A12,SNX1,SREBF1,STX3,SYT5,TAL1,TCF3,TCP1,TFRC,TIAM1,ZNF175,PXDN,PLA2G7,SHOC2,PPM1D,HIST1H2BJ,ATP6V0E1,FIBP,SPAG7,HDAC9,TM9SF4,ZNF623,TESPA1,TOMM70,ZBTB39,HIPK3,TRAP1,NDRG1,ARIH2,FAM3C,PPIE,SLC30A9,YME1L1,ZNF268,YWHAQ,RAB32,SF3B2,KIF3A,KLF8,IKZF3,RHOBTB3,SEPHS1,MAPKBP1,NFASC,DNAJC9,SF3B3,ACOT9,EID1,NBPF14,POU2F3,LETMD1,TMEM98,UBXN7,TIMM10B,ZNF330,CCDC59,PSAT1,HUNK,GEMIN4,EXOSC1,ASB3,CRIM1,WAC,NGRN,TRAPPC2L,SHC3,NEURL1B,MTRF1L,GNL3L,APTX,ANKRD49,GID8,FIGN,RCBTB1,CAMK2N1,PIP4P2,GALNT10,EMC7,BIRC6,KCTD16,TLDC1,CYP4F11,LY6G5B,RRAGD,PLEKHA2,TNS3,TUT1,NABP2,PVRIG,FN3KRP,ISOC2,RPAP2,CHD9,MPIG6B,BHLHB9,TXNDC5,NETO2,ATG10,SLC41A2,QRFPR,POLR1B,EIF1AD,AIFM2,UTP4,WDR73,ZC3H12C,ZNF766,ZNF486,PLXNA4,MTDH,MRRF,TJAP1,ESCO1,C1QTNF6,SLC25A26,TRMT61A,ANTXR2,ATP6V1G3,KLF17,RFTN2,ZBTB46,ZNF280B,TMEM120B,ZSCAN29,PPM1L,USP51,OR7D2,PLD6,TCTE1,ZDHHC20,FRMD3,TAB3,TTC9C,CAVIN1,TMEM105,SIGLEC15,ZNF780A,METTL2A,RILPL1,LHFPL4,LHFPL3,RPSAP58,NUDT19,FAM180B,TMEM236,GTF2H2C,NBPF10,GPR75-ASB3,NHSL2,SLC43A2,ZNF304,ACADS,NME6,ACAD11,FUT8,ATG2B,INTS2,KIAA1549,TMBIM1,NOTCH2,NOX5,PTPN9,CARTPT,ZER1,SERINC3,LILRB5,GABRP,SLC38A11,SLFN12L,LRRD1,VIPAS39,PPP1R8,SERTAD4,SKI,NSL1,TTL,MORC2,SLC2A5,POU6F1,HNRNPCL3,MYO1H,MED6,KCTD10,CDCA5,JAZF1,NDUFV3,MTERF1,ZNF740,ASPH,STMN4,SPTLC3,ENC1,CLCNKA,SLC9A7,RAB44,TRMT2A,ZCWPW1,PDE1A,TTPAL,UNC45B,WWP2,SLC16A7,CFAP410,SLC35A3,CSNK1A1,PNLDC1,KLHDC8A,AAGAB,SHISA5,KCNJ15,IGSF9B,TPM3,MON2,ZNF229,ZNF518A,BUB1,RANGAP1,FBXL4,CIRBP,MBD3,SSH2,MICAL2,FBXO31,EIF4E3,TRIP12,CCDC117,OPALIN,AURKB,SLC17A4,TMEM132B,TMEM179,RNF217,TMTC4,HSPH1,FARP1,TXNDC8,BTK,TBCE,SNTG1,UBN1,KIF6,ARHGEF3,CSPP1,NHS,CFAP97,RPA2,DESI2,MRPS18C,FAM120C,COA8,C1QTNF9,ZBTB26,CLDN4,TNS1,DCUN1D1,FANCM,SSR3,ZNF346,CDK11A,KDM5B,ZPR1,CXXC5,MTCH2,COL21A1,COX4I1,ALDH4A1,NLRP2B,NRXN1,MED28,LNPK,SERPINB1,APOLD1,ZNF442,TSPAN14,SLCO5A1,TRIM56,CALN1,PCDHA2,PCDHA3,MRPS5,ESYT3,NDRG3,KREMEN1,OTP,PCGF6,TXNDC2,RTL6,MEGF11,AP1M1,FAXC,NTNG2,ZNF347,MAFG,TMEM25,UBASH3B,CFAP300,GAL3ST3,DMRTC1,MYLK2,RWDD2A,MVB12B,FBXO21,STX1B,GPRIN1,SMYD4,CLNK,NIBAN1,RBM33,DUSP4,TSR2,EXOSC6,COLQ,DYNLL2,RAD51B,SLCO1A2,CREB1,PTCRA,RHBDL3,VASN,ZNF655,MAPK9,NACC2,RNF145,RIBC1,NEIL2,CCDC127,MOB3C,NFAM1,RELT,NOPCHAP1,CDYL2,NUDT16,JMY,HGSNAT,ZNF664,ZNF597,TMEM199,RNF165,LINGO2,FAM76A,MICU2,PDIK1L,DGKD,DGKH,TCEANC2,TCEAL8,LTO1,CAMKK2,HOXA1,NUP62,CACUL1,WDR17,UNC5B,CAMK2B,IFNLR1,NPAS3,USH1G,CCDC141,ZNF775,ZCCHC12,PLEKHA7,EMC10,DDX51,CBX7,PDLIM2,TRAPPC6B,LYNX1,ASAH1,PLA2G4D,NAT8L,HNF4A,ZNF445,BCDIN3D,BCL6B,ZNF778,RIMS4,ZNF169,PHC2,SCN5A,ING1,NKPD1,STIMATE,SIAH3,CENPS,MPRIP,USP17L2,FOXO3,BDH1,NMB,MS4A7,ENSA,SKIDA1,ZC3H14,PRUNE1,ARHGEF10L,NYAP2,COL6A6,SYNCRIP,RBM48,FNBP1,ABI1,PFKFB3,ADAMTS17,FOSB,PTPRS,ARVCF,LAS1L,MFN2,RALB,MARK3,SLC36A1,ATF6,PSD4,CHIC2,MAFK,MAN1B1,STAMBPL1,CACNA1C,FERMT2,ZNF569,SLC16A14,ATP13A3,CYBA,POU2F2,UNC13A,SGTA,COL20A1,PPARA,PPM1F,DNASE1L1,TMEM154,REEP1,MED13L,ESPN,GNG4,NTNG1,KAZN,TNR,EFCAB2,RGS8,PLEKHM3,ABCC5,USF3,SCAP,IP6K2,GPNMB,SETX,CDON,KCTD13,YPEL2,ZC3H4,TBC1D22A,SYN3,RPS6KA6,NBN,ZNF706,ZFAND6,AGBL4,ZNF652,PRR11,CLTRN,MBD5,TIGIT,RXYLT1,HABP4,TMEM50A,HSPB8,SESN1,SH3BP4,TFCP2L1,ULK2,GARRE1,CEP350,PJA2,RHOBTB1,SV2A,HELZ,N4BP2L2,FSTL4,COLGALT2,ANKLE2,NCAPD3,FEM1B,SEC31B,DNM3,PGM3,TRAF3IP1,KIF26A,RRP7A,COL5A3,PCF11,CDC40,GLOD4,CAB39,POLR3K,GOLM1,LGSN,RSF1,MYOZ2,SLC25A37,HOXC10,BAIAP2,ATXN2L,ATAD2B,WDR5,SAMD9,GATAD2A,WDR55,ODR4,VPS37C,RNF220,OGDHL,RNLS,SLC35C1,METTL2B,CHDH,CHST11,SELENOS,EMC3,ANLN,ETAA1,OTULINL,HOXD8,CTPS2,UBQLN4,FEM1C,LHX9,CDC42SE2,PAPPA2,JPH2,FAM219B,PAIP2B,CBX8,MAVS,XPO5,ARHGAP31,ALPK3,HCN3,ZBTB4,SLC7A14,KIAA1614,EPG5,SNTB1,PMAIP1,CCAR2,RHOU,TMEM35A,TICAM2,ZNF711,TIA1,FAM204A,EGLN3,PERP,TCTA,TPK1,NSD1,ZNF106,TTC31,SMAP2,SMURF2,GAREM1,IPPK,ILRUN,FNDC4,C1orf116,NDUFAF5,TSPOAP1,WNT2B,ICE2,SAP30L,GTDC1,CARF,PREX2,MAP6D1,PSME3IP1,UXS1,NAA50,PHAF1,FRK,GSK3B,KCNJ6,LYN,MAS1,PDK4,MAPK1,CHMP1A,PTPN4,RBM4,SELENOW,SHB,UBE2G1,ZNF37A,ULK1,SLC4A7,RDH16,CFLAR,CDKL2,DMD,EIF2S1,PRKAR2A,SLC1A1,ARHGAP1,ARL3,KIF1A,HOXC6,FOXK2,MEOX1,NFATC3,SCO1,SYNGR1,ARHGAP29,ENTPD4,PDIA4,GABRE,KCNJ9,MECP2,MYO1E,PLK1,RORC,THRAP3,NUP153,GPR37,UBE2K,PDCL,ROCK1,SLC20A1,PIGK,SOS1,CERT1,NUTF2,MELTF,CEPT1,NEUROD2,PDGFRA,PIN4,POU3F3,POU4F1,DPF2,TFDP2,TYRO3,TRIM38,MTHFS,POLR3G,MCAM,KIF1C,MSMO1,SNTB2,SYNGAP1,LMAN2,RAB40B,UQCR11,DSTN,SUMO3,ZBTB25,HNRNPUL1,TUSC2,DLEC1,GNA12,PLA2R1,MLYCD,KCND2,TLL1,TRPC5,SEC61A1,DSE,KCNK2,ZNF160,CDKL5,ACADSB,PCNX4,HNRNPA3,AKAIN1,LRRC7,PDXK,OR6J1,USP19,CEP63,H3Y1,TMEM225,PRPF40B,RPL7,ZFAND2A,P4HA2,TPM1,KPNA5,DCBLD1,CA5A,DMRTC1B,ANHX,PLD5,EHF,B4GALT1,TNPO2,DMAC2L,PTCHD4,SPECC1,PNMA8C,OR56A1,TADA2A,IGSF3,INPPL1,GRK2,APLP2,BRS3,CLCNKB,GLDC,NCF2,SLC11A1,CNTF,CACNA1B,CACNA1E,GABRA4,HTR1D,PRRG1,RPL22,RPL37,RECQL5,OR1B1,OR10H5,OR51D1,OR51G2,CHMP3,ZNF484,RNF187,MTX3,DDX60L,SNX30,HYKK,PAK4,LGR6,SH3PXD2B,FAHD1,SERF2,ZNF805,RPS26,CPLX3,SPN,CYB5RL,ITPRIPL2,PTGFR,CCNI2,INTS6,PAQR3,CAST,SLC6A3,DPH5,HNRNPUL2,ARHGEF28,BICC1,MRGPRF,TIFAB,ADAM8,ADAR,NAV2,NBEAL1,GALNT9,PPM1M,ELF4,FLNC,STXBP5,GSPT1,EPOP,MICAL3,GPR107,NIPA1,ZNF534,UBAC2,ZNF814,CCDC85C,C11orf96,ANKRD34C,PLEKHD1,GYS1,STAU2,TNFAIP8L1,NDUFB4,SLC6A2,TSFM,SEC23B,DPY19L3,PNPLA5,NIT1,RBM14-RBM4,MYO16,ST20-MTHFS,ACTG2,MBD1,RAB43,ISY1-RAB43,OCLN,ARFGAP2,FOXM1,ENTPD3,PASK |
| **miR-362-3p** | ATP1B3,ETFDH,EVI2A,GSTM5,ABLIM1,MGAT5,PTPN1,UBE2V1,AAK1,POT1,ABCA12,GPR78,ZNF562,ADRB3,ALDH1A3,ABCC6,ARNT,ATP1A3,C6,CA8,CD19,CFL2,CHEK1,CLTB,CPE,CRK,CSNK1D,CTGF,CYLD,DDB1,DMXL1,ELK1,ETS1,F2RL1,FGF9,FOXL1,GCK,GDNF,NPBWR1,GPR21,RAPGEF1,HLF,HMGB2,HSPA4,IGBP1,IL5RA,ITPKB,CD82,KCNA4,KCNJ3,KCNJ12,KIF5C,LFNG,MATN1,MC2R,CD99,MOBP,NDUFS1,NDUFS2,PAX3,PCDH7,PDK3,PHEX,PHKA1,PIK3R1,PLRG1,MAPK6,PRKX,PTPN3,RAB1A,RAP2B,RDX,SBF1,SCN4A,SRSF4,SLC1A4,SLC12A2,SLC22A4,TCF15,TCF20,TMBIM6,TFCP2,NR2C2,TRAF5,TNFSF4,UCHL3,UGT2B4,UGT8,WNT9A,WRN,SF1,SLBP,PTP4A2,NCOA3,CHAF1B,HIST2H2AA3,BHLHE40,KCNK5,LAMTOR3,RNMT,ADAM9,WISP1,BSN,KYNU,TBRG4,B4GALT6,B4GALT5,STOML1,TBPL1,BCAR1,GNA14,CEP135,WSCD2,STARD8,RIMS3,SPCS2,TATDN2,MED24,FIG4,SV2B,ATP9A,TSPAN2,TSPAN1,ARL4C,SORBS3,SMNDC1,LILRB2,CCT4,NFAT5,LILRB1,WDR3,PPARGC1A,PNRC1,CPSF6,CACFD1,PRSS23,IL1RAPL1,NUDT3,PDCD10,CBX3,CEP162,COBLL1,WDR37,KIAA0907,WDTC1,WAPL,HIC2,PHF8,NUP205,MESD,MDN1,SMCHD1,ARHGEF12,SRGAP2,SIK3,ZDHHC17,RHOQ,DAAM2,ACAP2,TNPO3,LPAR3,GABARAPL3,BAMBI,REXO2,LSM14A,SERBP1,ZBTB20,FBXO9,TIMM10,STK36,TNFRSF21,VENTX,BMP10,SLCO1B3,FHOD1,TMOD2,DONSON,DNTTIP2,EHD3,EHD2,ASCC1,TUBD1,CCDC174,CHST15,RHCG,TRMT112,MPP6,SYT17,PHAX,CRLS1,38412,BNC2,SNRK,SEMA4C,NOP14,MDGA1,MTIF2,TMOD1,STK24,SPOPL,KCTD21,MPZL1,FLYWCH1,RTL4,HIP1R,BCL2L14,C1orf74,C3orf62,EMP2,ESRRB,ZSWIM4,AHNAK,PCGF5,GRM4,CDH13,SENP1,ATRIP,AAR2,EPC1,EIF4E2,TP53,AOC3,NBL1,LIAS,PTGDR,GNA13,TMEM108,MAB21L4,LRRTM4,PGAP2,TANGO2,DBNL,HMOX2,SLC22A23,C2orf49,RMDN1,OTOF,TTC23,ZNF585A,ERBB2,ATL3,C3orf14,MAN2B2,CDCA3,PEX5,PRDM5,ABLIM3,VASH2,DEFB134,PALS2,ZNF500,EPHA4,SPRY3,ST8SIA5,ZKSCAN5,SEMA3F,ABHD18,ODF4,FAM90A1,GDPD5,DMRTA2,WDR75,VPS25,CLIP2,JAGN1,NAA11,ZNF514,CEP89,TNS4,DTNA,PDGFB,EAF1,TBCK,PPP1R3F,TP53INP1,SLC2A13,BORCS5,PTPN7,COL13A1,PTPRC,PTPRD,SGSM1,SYN2,HNRNPLL,HINT3,AKAP7,PPT2,A1CF,PHETA1,CDH24,ZSCAN20,PRRT2,DDIT4L,METTL21A,RDM1,ZNRF2,FAM219A,RNF185,PARS2,PRXL2B,UBXN10,KLHL40,UGT3A1,TMTC2,GPC2,PSMC4,GPR156,LACC1,RC3H1,DCUN1D3,ARHGEF15,CES4A,OGFOD3,UBE2D2,TMEM65,ZBTB41,CA13,ESRRG,FAM3B,POGZ,MARVELD2,C19orf38,TLR6,AGAP2,RUBCN,PDPN,CARD8,PRL,GRID2IP,CLUH,ALDH1L2,ZBTB16,DCAF5,VEZF1,LIMS2,LIMD1,VPS41,GALNT5,TOMM20,SGSM2,ENPP4,ERP44,ANAPC13,PIGV,TMEM70,HIF1AN,RBM23,PBRM1,LRIF1,WDR33,COPRS,PAG1,LMO3,CTTNBP2NL,DHTKD1,AGPAT4,STARD7,STOX2,S100A14,METTL14,ZNF2,ENPP5,ZNF148,HIPK2,FUNDC2,YIPF2,DERL1,SH3TC2,PLEKHF2,TUT7,IGFLR1,METTL8,NANOG,DLST,GNA15,HMGCS1,INHBA,KIF3C,MGAT3,QSOX1,RCN2,ROS1,SAT1,SLC9A2,SORL1,THBS2,CDK5R2,RAB27B,CHST3,AKAP6,BRAF,TOP3A,KIF3B,KCNK6,SPTLC2,NR5A1,PHF2,TIE1,NAMPT,RBM5,GDF11,PLIN3,DCAF7,CAPRIN1,PEG3,PIK3CA,ZNF230,NOVA1,TREH,SLC7A8,CLCF1,DIO2,NDUFAF4,CRIPT,MRPL22,CDIN1,NIPAL3,RBMS3,NCBP2L,KANSL3,TPRA1,SPAG11A,SAMHD1,RGMB,SYT16,SMIM38,ZBTB42,SEMA4D,ZNF677,OR2AG2,DPYSL3,FMO5,F13A1,NDP,PECAM1,HEXA,ADRA2B,DRD1,ARHGEF37,TPD52L3,KLK2,SLC6A19,OR2M3,OR6C75,SOGA3,PLIN5,TNFRSF1A,TCF19,MEX3A,ZNF578,SERPINA10,CABLES1,PIRT,PRRT4,ADCY9,THAP5,TMEM151B,LRRC27,FKBP5,TMEM64,ABL2,VIT,TMEM178B,TPBGL,SIGLEC9,CCDC169-SOHLH2,MICOS10-NBL1,TMEM235,IRF6,HNRNPDL,CA12,DCP2,WDR20,HLA-DPA1,SEPTIN7,HINFP,WNT5A |
| **miR-548-5p** | FOXP2,ZNF518B,NUP98,FSIP1,ZBTB9,NSMCE2,MZF1,ANKEF1,DHDDS,SYT15,PHF14,POFUT1,FGD6,KIDINS220,ADCY1,ABCG8,PAK2,FBXO8,TRPS1,MEI4,SYNE3,ASH1L,WIPF1,OR2J3,KATNAL1,DIPK1C,SAMD12,SIRT1,SRSF10,PPP6R2 |
| **miR-24-1*** | SLITRK1 |
| **miR-33a** | ABCA1 |
| **miR-7641** | ADCYAP1,ANG,CDK4,CDKN3,DRP2,EIF4E,GLG1,GOT1,GSTM3,IGF1,IL6R,ORC1,PLSCR1,MAPK1,REL,SCN7A,SCO1,SIM2,SIX1,SNTB2,SPIB,TFDP2,TSPAN6,USP1,XBP1P1,ZSCAN9,SLC30A4,ARID1A,DGKE,TNFSF14,CDKL1,USP14,TRIP4,CIAO1,AKAP6,CYTIP,SOCS5,CEP170,TRIM66,GNE,TRIM10,SERF2,ZNF256,COQ7,IGSF6,TLK2,BTN3A2,HSPA4L,ZNF652,CHSY1,DIP2A,PPWD1,NPTXR,PHLDA3,TMEFF2,MTO1,TECPR1,POC1A,RTTN,HINFP,ZNF451,PYGO1,TMEM251,HEYL,RNF115,P2RY10,UFM1,MYOZ2,PAQR5,MTMR10,BANK1,FKBP14,UEVLD,ETNK1,HIF1AN,SAR1A,UGGT1,CABP4,JPH2,TAOK1,SLC7A14,TGIF2,EXO5,GNPNAT1,ZNF665,RNF122,DNAJC22,CHD9,RAB11FIP1,ORAI2,WDR26,ZNF436,YIPF5,TSPAN14,QRFPR,YIPF4,BRMS1L,PPP1R15B,ZNF799,TIMM29,ZNF845,MYLK3,SYAP1,FAM83F,PWWP2A,FLYWCH2,COX20,GINM1,FSD2,KRBA2,CMBL,STARD4,ZNF786,DOCK11,PUS10,KLB,ZNF320,ALG14,CC2D1B,EPGN,MFSD8,NUDT7,ARL10,ZNF677,MOGAT3,FAM71F2,ARL5C,FAM180B,TMEM215,GOLGA7B,GTF2H5,POTEG,PIM3,POTEM,SHISA9,LRRC3C,PPP5D1 |

**Table S2: Unique transcription factor genes targeted by the miRNAs from the 2 datasets obtained from POMA analysis**

| **miR** | **Unique transcription factor genes (UTP>0)** |
| --- | --- |
| **let-7b-5p** | CBFB,CHD3,CTBP2,E2F2,EP300,GTF2I,HIST1H1C,HES1,HSF2,IGHMBP2,NFATC1,NFKBIA,PCBP2,POLR2H,RXRB,SALL2,SMARCA1,SMARCA4,SP100,TCOF1,NR2E1,UBE2I,ZNF3,ZMYM2,BRPF1,TRIM24,COPS2,SOX13,SCML2,RAI1,DDX20,PMF1,SCAF8,PDCD11,PPRC1,ATG4B,ZCCHC11,CARHSP1,MKRN2,PLXNB2,CIZ1,POLR1A,AHCTF1,TOX3,UHRF1,SFMBT1,TAF9B,CHRAC1,TERF2IP,MIER2,ANKZF1,TRMT1,YEATS2,POLR3B,ZNF687,DNAJC1,GZF1,CCDC71,LIN28A,ZNF606,NAA15,MED25,CDCA7,DHX57,L3MBTL4,MFSD3,TOE1,HIPK1,ZNF841,HOXA11,TEAD3,CSDE1,PRICKLE3,CALCOCO1,ISL1,WWTR1,FHL1,PHF20,LIMA1,TARBP1,DLX3,HMG20B,ZNF76,HDAC4,HLTF,PICALM,TEAD2,SART3,XAB2,TOP2B,NFKB2,SMARCA2,RNF13,ATRX,ZNF343,RBBP9,CDIP1,ZNF184,JAK2,ERMP1,HIRA,PLEK2,PNN,TCFL5,TRIB3,STAG2,FMR1,HTATSF1,VGLL1,KLHL4,TAF7L,KLF5,RGCC,CENPT,ZNF423,TSC2,FOXF1,HERC1,ZDHHC2,RELB,SNAPC2,PPP2R1A,LSR,SP4,HOXA5,HOXA13,TAX1BP1,POU6F2,CREB3,GLIS3,KAT2A,RCVRN,CREBL2,GTF2H3,TDP2,TULP1,PRDM13,EXOC2,DLX2,STAG1,KLF7,NR4A3,SMAD9,KBTBD7,ZC3H7A,TAF1L,PKN1,OPTN,ATF1,HOXC13,HOXC11,HOXC12,ZNF45,RNF113A,BMP4,SLC2A4RG,TRIP10,BMP2,IRF3,ZBTB1,CDKN1C,ZNF428,CDX4,PPARG,TRIM47,ZMYM5,RFXAP,ZNF414,GSC,SOX5,PLXNC1,MED4,ACTL6A,POLR1E,GCM1,TGS1,TRIM29,HNRNPD,TDG,CUL4A,JDP2,GTF2A2,TGFB1I1,MYOCD,BDP1,SHPRH,RBAK,NONO,ZNF185,UHRF2,CDKN2A,ANKRD1,NPAT,MKX,NR3C2,RNF144A,FLI1,ZFP36L2,DAB2,DEPTOR,GRHL3,EIF5B,CSRP1,ZYX,DEDD2,FAM189B,MFSD12,RPS6KA4,ATXN7L2,EN1,ELF3,RNF25,IFI16,TIPARP,MST1R,EBF1,TLX3,CREBRF,SNAPC3,CDX2,PCBD1,STAT6,TSC22D4,SMAD3,PHB,PRRX2,NFKBID,IRF2,ZNF30,MN1,TBC1D10B,LDB2,GSX1,ZNF768,ZNF296,ZNF524,ZNF274,MLLT3,HOXC5,ZNF621,TIGD3,GLIS1,ATR,DDIT3,DRAP1,ETV4,ANAPC2,FOXC2,NFATC2IP,ZBTB38,IRX3,BOLA1,TIGD2,ZNF571,NRIP1,SETD2,LDOC1,SATB1,CRIP2,MTA1,NKX2-5,RIPK4,ALYREF,TRIM52,KNTC1,SMYD3,NR2F2,TAL2,ZNF397,ZNF385C,ZFP69B,ZC3H6,SP6,PNRC2,ZNF136,ZNF418,MAML3,SLC39A10,ZNF785,ZNF165,TOPORS,TEAD4,HMGN5,ZNF251,ZNF813,PLXNB3,CNOT7,ZNF521,TOX,ZFP2,INF2,FANK1,FOXO6,MAFB,PRR3,TRIM13,CRIP1,MLLT11,MXD3,IRF9,ZBED1,ZNF579,ZNF879,KIAA0040,RBM14,STON1,ETV5,ZNF718,ZNF432,ZBED6,ZNF587B,PCGF2,ZNF670 |
| **let-7c-5p** | CCNC,DLX4,DNMT1,EWSR1,HOXD1,JARID2,RARB,TAF5,TTF1,COPS3,SNW1,RFX6,HES5,MBTPS2,PRKDC |
| **miR-3195** | MSX2,VDR,ARC,CASZ1,SIX5 |
| **miR-4443** | ARHGAP35,ZNF69,TRIOBP,CSDC2,TSG101,UBE3A,MIS18BP1 |
| **miR-4459** | CBFA2T3,DR1,EGF,NFATC2,RBBP8,SOX12,WNT8B,ZNF212,MED16,KLF2,ZCCHC4,SERTAD3,HP1BP3,ZNF280C,ZFP64,HIF3A,KLHL25,PAPOLG,ZNF747,ZNF329,ZNF394,ZNF527,ATOH8,LHX4,ELMSAN1,ZNF764,ZNF101,ZNF554,ZNF583,ZNF780B,ZBTB49,ZNF549,HMX3,ZNF850,ZNF788,ZBTB8A,ZBTB8B,ZNF783,ZNF891 |
| **miR-4485-5p** | KDM5A,VHL,TAF1B,PIAS2,STRN3,ZSCAN2,KCMF1,GATAD1,GPBP1,ZNF419,ZBTB3,ZNF384,ZFP82 |
| **miR-4505** | TAF1,MLX,DMRT2 |
| **miR-4530** | PTCH1,ZNF154,ZNF208,MIER1,HES4,ZNF34,DZIP1L |
| **miR-494-3p** | ATF3,BMI1,SLC26A3,ZBED2,BCLAF1,RNF19A,TRIP13,MCM2,XRCC5,ANKRD10,NFKBIB,PIAS4,DNAJC2,NEIL3,NOP2,SRSF9,KDM3A,TNFAIP3,KHDRBS1,SRSF6,RNF114,MED20,SNRPB,HNRNPR,POLR3F,MEIS2,DDB2,PWP1,CNPY3,LRPPRC,SSB,CERS5,PARN,LMO4,MEIS1,AFF3,LYAR,BTF3,ZMAT2,INTS4,U2AF1,CPSF4,LARP4,RBBP4,FUBP1,SAP30,NFIL3,ZNF22,ZBTB43,APEX2,CAMTA1,ZNF131,AFF1,STAT5B,ZDHHC13,SIAH2,SS18L1,TCEA1,XRCC6,ZNF248,WDHD1,ZNF830,ZNF254,PPP3R1,ZBED5 |
| **miR-6088** | AIRE,SP110,YWHAB,HIST1H2BG,C1orf61,RBM27,TAF1D,CERS4,ZNF429 |
| **miR-6089** | NKX2-2,GREB1,KCNIP3,ZNF557,ZNF491 |
| **miR-6127** | HPCAL1,POLR2F,SP2,ZIC2,ZNF70,DPF1,IKBKG,HOXB13,UBR4,HES6 |
| **miR-642a-3p** | FOXN3,SSX2,LARP4B,ZNF257,SMYD1,YY2 |
| **miR-6740-5p** | DYNLL1,ANKS1A,ZMAT1 |
| **miR-6780b-5p** | EMX1,HOXD3,HOXD13,JUND,NAB2,RREB1,VPS72,SMYD5,RBPJL,GLIS2,ZNF467,ZSCAN25,ANKRD45,IQSEC3 |
| **miR-6879-5p** | EVX1,TBC1D2B,ZMAT4,FBXO41,ZNF322P1 |
| **miR-7641** | PLSCR1,TRIP4,TRIM10,ZNF436,ZNF845,ZNF786 |
| **miR-8069** | RFX1,LHX6,ZNF853,ZMIZ1,HOPX |
| **miR-939-5p** | ARRB1,ASCL2,HLX,MYBL2,UBTF,PER3,MSC,GTPBP1,HDAC5,ZNF561,CREBBP,HDAC7,NCOA1,TFAP2C,CDC6,PRPF6,HOXA6,LZTS2,TRIM3,PHF1,ZBTB47,HOXB8,KDM4B,ILF3,ZNF236,KRBA1,LMO7,CARM1,NFKBIZ,GTF3C5,ZNF318,CERS6,JUN,TAF7,NDUFA13,USP7,C11orf95 |
| **miR-101-3p** | APP,RHOA,ATM,NKX3-2,PRDM1,CDK8,CDKN1A,ELAVL2,EZH2,FOS,HNRNPAB,HNRNPU,MET,MYCN,NOTCH1,PIM1,RORA,SMARCD1,TAF13,ZEB1,TFAP4,THRB,UBE2B,ZNF124,ZNF207,ZNF223,ARID1A,EEA1,CBX4,CTR9,BZW1,DAZAP2,TSC22D2,ZC3H11A,MBNL2,SUB1,SNRNP27,DIDO1,NUPL2,KLF12,SUZ12,TNRC18P2,CERS2,ZBTB7A,NLK,KDM3B,SIX4,PSPC1,ZNF654,TBX20,ZNF490,PRDM16,FOXP4,ZNF480,ZNF645,ZDHHC15,ZNF567,ZNF431,CHAMP1,NANOGNB,LIN28B |
| **miR-142-3p** | LPP,APC,CDKN1B,GFI1,HMGB1,HMGA1,HOXA10,IRF1,NAP1L2,PSMD11,PSMD12,NR2C1,ZNF217,CUL5,ROCK2,MORF4L2,ATF5,ZMYND8,YOD1,DEPDC1,ZNF701,RNF20,ZNF608,ZNF529,WIZ,MESP2,HSFY2,ZFPM1,ZNF676,ZNF678,HES2,ZNF99,ZIC4,ZBTB10,RBM39,ZNHIT6,ZXDB,HMGB3,ZNF501,PGR,ZNF107,ZNF286A,ZNF717,ZMIZ2,ZNF773,TSHZ1,HNRNPK,CSRNP2,SCRT1,SOX6,BRWD1,TBX5,PPARGC1B,ANKRD54,ATOH7,TRAF6,ZNF114,DMBX1,TSHZ2,ZNF776,CERS3,GAS7,CSRNP3,MGA,ZNF181,ZNF324B,NRG1,MYT1L,KAT6A,ETV3L,OTX1,ZHX3,TECPR1,KLF13,MYEF2,PHF21A,BBX,NEUROG3,E2F5,AFF2,MYC,NDN,MAPK3,SRSF3,SSRP1,MED1,DLX6,ERBB4,GLI2,SMAD4,NFE2,NFRKB,RORB,ZNF81,ZNF180,PREB,KLHL21,ZNF738,IKZF5,ZNF320,ESR2,TGFB1,ZNF182,EVX2,AMOT,TP63,UHMK1,YAP1,MEFV,CCNH,TCF4 |
| **miR-29b-3p** | BCL2,CCNA2,DNMT3B,FBN1 |
| **miR-32-5p** | GATA6,HIVEP1,HOXC8,FOXN2,SMAD7,MDM2,CNOT4,PAWR,PAX9,PTEN,RAD21,SNRPD1,SOX4,TMF1,ZNF17,ZNF157,ZNF224,MBD2,KLF4,ZSCAN12,SERTAD2,AP5Z1,SUPT7L,TOB1,CTDSPL,ZNF267,PRMT5,IKZF2,PAXIP1,RBFOX2,MYCBP,PURG,BAZ2B,ERGIC2,BCL11A,BTBD2,ATF7IP,OTUD7B,ZNF695,GATAD2B,ARID1B,ZNF398,KLHL14,ZNF492,ZNF317,PCBD2,ZNF594,GLYR1,ZIC5,LIN54,ZNF417,ZNF98,ZNF383,MIER3,ZDHHC21,ZNF772 |
| **miR-342-3p** | FOSL2,ZNF548,ABL1,ATF4,CALR,MLLT6,RFX3,SREBF1,TAL1,TCF3,ZNF175,ZNF623,ZBTB39,HIPK3,ZNF268,IKZF3,CCDC59,ASB3,APTX,ANKRD49,TUT1,CHD9,ZNF766,MTDH,MRRF,KLF17,ZNF280B,ZSCAN29,ZNF780A,GTF2H2C,ZNF304,KIAA1549,NOTCH2,PPP1R8,SKI,POU6F1,MED6,JAZF1,ZNF740,ENC1,ZNF229,MBD3,SSH2,UBN1,RPA2,ZBTB26,ZNF346,KDM5B,ZNF442,TRIM56,OTP,PCGF6,ZNF347,MAFG,DMRTC1,RAD51B,CREB1,NACC2,NEIL2,JMY,ZNF664,ZNF597,TCEAL8,HOXA1,NUP62,NPAS3,ZNF775,CBX7,HNF4A,ZNF445,BCL6B,ZNF778,ZNF169,ING1,MPRIP,SKIDA1,ZC3H14,LAS1L,ATF6,MAFK,ZNF569,POU2F2,PPARA,MED13L,TBC1D22A,ZNF706,ZNF652,POLR3K,RSF1,WDR5,GATAD2A,HOXD8,CBX8,NSD1,SMURF2,CARF,MAPK1,HOXC6,FOXK2,MECP2,RORC,THRAP3,UBE2K,ROCK1,POU3F3,POU4F1,DPF2,TFDP2,TRIM38,HNRNPUL1,HNRNPA3,ANHX,EHF,TADA2A,ZNF805,ELF4,ZNF534,ZNF814,MBD1,FOXM1 |
| **miR-362-3p** | MGAT5,ZNF562,CRK,DDB1,FOXL1,HLF,PAX3,PLRG1,TCF15,TCF20,TFCP2,NR2C2,SF1,NCOA3,BHLHE40,TBPL1,RIMS3,MED24,CCT4,NFAT5,PNRC1,CBX3,HIC2,PHF8,ZDHHC17,REXO2,ZBTB20,STK36,DNTTIP2,ASCC1,ESRRB,EPC1,TP53,NBL1,ZNF585A,ERBB2,PRDM5,ZNF500,ZKSCAN5,DMRTA2,VPS25,ZNF514,ZSCAN20,RC3H1,ESRRG,POGZ,ZBTB16,VEZF1,LIMD1,HIF1AN,PBRM1,LMO3,ZNF2,HIPK2,TOP3A,NR5A1,PHF2,RBM5,PEG3,ZNF230,ZBTB42,TCF19,ZNF578,THAP5,IRF6,WNT5A |
| **miR-548-5p** | FOXP2,NUP98,ZBTB9,MZF1,TRPS1,ASH1L,SRSF10 |

**Table S3: List of miRs biomarkers for AMI/ACS from previously published data**

| **S.No** | **MicroRNA** | **Marker** | **Reference** |
| --- | --- | --- | --- |
| 1 | miR-1 | Diagnosis | Ai J 2010; Wang GK 2010; Li YQ 2012; Long G 2012; Oerlemans M 2012; Liu X 2015 |
|  |  | Prognosis | Widera 2011; Gidolf 2013; Grabmaier 2017; Goldbergova 2018; Hromadka 2019; Mayer 2019; Su T 2019 |
| 2 | miR-21 | Diagnosis | Oerlemans M 2012;Olivieri 2013; Darabi F 2016 |
| 3 | miR-26a | Diagnosis | Ren J 2013; Li C 2015 |
| 4 | miR-133a | Prognosis | Eitel 2012; De Rosa 2017 |
| 5 | miR-208b | Prognosis | Lv 2014; Gacon 2018 |
| 6 | miR-499 | Prognosis | Gacon 2018; Gidolf 2013 |
| 7 | miR-375 | Prognosis | Devaux 2013a; Gacon 2018 |
| 8 | miR-34a | Prognosis | Matsumoto 2013; Lv 2014 |
| 9 | miR-652 | Prognosis | Pilbrow 2014 |
| 10 | miR-328 | Prognosis | He 2014 |
| 11 | miR-134 | Diagnosis | Li C 2013; Tong KL 2018; Wang KJ 2016 |
|  |  | Prognosis | He 2014; Gacon 2018 |
| 12 | miR-145 | Prognosis | Zhang M 2017; Dong 2015 |
| 13 | miR-197 | Prognosis | Schulte 2015 |
| 14 | miR-223 | Prognosis | Schulte 2015 |
| 15 | miR-122 | Diagnosis | Li X 2015 |
|  |  | Prognosis | Cortez-Dias 2016; Gacon 2018 |
| 16 | miR-133b | Prognosis | Cortez-Dias 2016 |
| 17 | miR-30a | Diagnosis | Long G 2012 |
|  |  | Prognosis | Maceijak 2018 |
| 18 | miR-19a | Prognosis | Mayer 2019 |
| 19 | miR-126 | Diagnosis | Xue S 2019 |
|  |  | Prognosis | Schulte 2015; Mayer 2019 |
| 20 | miR-186 | Diagnosis | Wang KJ 2016 |
|  |  | Prognosis | Li Z 2019 |
| 21 | miR-142 | Prognosis | Guo 2020 |
| 22 | let-7b | Diagnosis | Long G 2012 |

**Table S4:** **Differentially expressed miRs obtained from GSE148153 dataset after sub-analysis, in comparison to the initial analysis. Data are expressed as log fold change (log_2_FC) and -log_10_ p-value**

|  | **Sub-analysis (without female)** | | | **Initial analysis (female included)** | | |
| --- | --- | --- | --- | --- | --- | --- |
| **miR ID** | **log2FC** | **neg log10 p-value** | **Expression** | **log2FC** | **neg log10 p-value** | **Expression** |
| miR-494-3p | 2.329 | 5.730 | Upregulated | -2.338 | 6.939 | Downregulated |
| miR-7641 | 1.098 | 3.497 | Upregulated | -1.143 | 4.951 | Downregulated |
| miR-3195 | -3.193 | 4.383 | Downregulated | 2.851 | 4.237 | Upregulated |
| miR-4485-5p | 1.537 | 2.821 | Upregulated | -1.596 | 3.564 | Downregulated |
| let-7b-5p | -1.337 | 3.400 | Downregulated | 1.297 | 3.836 | Upregulated |
| miR-21-5p | -0.349 | 4.309 | Downregulated | 0.826 | not significant | Upregulated |
| miR-6088 | -1.324 | 3.101 | Downregulated | 1.0774 |  | Upregulated |
| miR-939-5p | -0.919 | not significant | Downregulated | 1.036 | 3.376 | Upregulated |
| miR-6875-5p | 0.923 |  | Upregulated | -0.919 | 3.474 | Downregulated |
| miR-6740-5p | 1.229 |  | Upregulated | -1.250 | 3.162 | Downregulated |
| let-7c-5p | -3.802 |  | Downregulated | 3.515 | 2.883 | Upregulated |
| miR-4505 | 1.985 |  | Upregulated | -2.575 | 2.764 | Downregulated |
| miR-6780b-5p | 0.923 |  | Upregulated | -0.885 | 2.757 | Downregulated |

**Table S5: Relationship between miRs and their target gene expressions involved in vital signaling pathways in AMI obtained from GSE148153 and GSE24591 datasets.**

| **miR ID** | **miR expression** | **Target gene** | **Target gene expression** | **Pathway** |
| --- | --- | --- | --- | --- |
| miR-101-3p | Down regulated | PRKCA | Up regulated | Cardiac hypertrophic response |
| miR-6875-5p | Down regulated | ATP2B1 | Down regulated | Calcium regulation in cardiac cells |
| miR-6875-5p | Down regulated | PIK3R3 | Down regulated | PI3K-AKT-mTOR signalling pathway |
| miR-342-3p | Up regulated | PTCH1 | Up regulated | Glypican-3 network |
| miR-362-3p | Down regulated | PTCH1 | Up regulated | Glypican-3 network |
| let-7c-5p | Up regulated | IL6 | Up regulated | Adipogenesis |
| let-7b-5p | Up regulated | IL6 | Up regulated | Adipogenesis |
| miR-142-3p | Down regulated | IL6 | Up regulated | Fibrin complement receptor-3 signalling pathway |

**Table S6: Association of variants in target genes with major phenotypes related to CVD obtained from the knowledge portal for CVD**

| **Target gene** | **SNP/SNV** | **Phenotype** | **Group** | **P-value** |
| --- | --- | --- | --- | --- |
| PRKCA | [rs1801689](https://cvd.hugeamp.org/variant.html?variant=rs1801689) | LDL cholesterol | Lipids | 7.14E-103 |
|  | [rs8178824](https://cvd.hugeamp.org/variant.html?variant=rs8178824) | Total cholesterol |  | 3.11E-53 |
|  | [rs1801689](https://cvd.hugeamp.org/variant.html?variant=rs1801689) | Triglycerides |  | 1.76E-48 |
|  | [rs8178824](https://cvd.hugeamp.org/variant.html?variant=rs8178824) | Non-HDL cholesterol |  | 2.22E-34 |
|  | [rs75003668](https://cvd.hugeamp.org/variant.html?variant=rs75003668) | Dyslipidemia |  | 1.14E-09 |
|  | [rs7211380](https://cvd.hugeamp.org/variant.html?variant=rs7211380) | Creatine kinase | Cardiovascular | 2E-32 |
|  | [rs55960558](https://cvd.hugeamp.org/variant.html?variant=rs55960558) | Diastolic blood pressure |  | 1.28E-09 |
|  | [rs9892651](https://cvd.hugeamp.org/variant.html?variant=rs9892651) | Left ventricular end-systolic volume (BSA-indexed) |  | 2.1E-09 |
|  | [rs4380082](https://cvd.hugeamp.org/variant.html?variant=rs4380082) | Systolic blood pressure |  | 3.21E-09 |
|  | [rs112699970](https://cvd.hugeamp.org/variant.html?variant=rs112699970) | Pulse pressure |  | 1.15E-08 |
|  | [rs12449326](https://cvd.hugeamp.org/variant.html?variant=rs12449326) | Heart rate |  | 3.54E-08 |
|  | [rs12601850](https://cvd.hugeamp.org/variant.html?variant=rs12601850) | Height | Anthropometric | 2.41E-27 |
|  | [rs11655080](https://cvd.hugeamp.org/variant.html?variant=rs11655080) | Waist-hip ratio |  | 7.28E-16 |
|  | [rs9912468](https://cvd.hugeamp.org/variant.html?variant=rs9912468) | Waist-hip ratio adj BMI |  | 1.23E-12 |
|  | [rs79973219](https://cvd.hugeamp.org/variant.html?variant=rs79973219) | BMI |  | 3.44E-08 |
|  | [rs9912468](https://cvd.hugeamp.org/variant.html?variant=rs9912468) | QRS interval | ECG traits | 2.19E-21 |
|  | [rs9909004](https://cvd.hugeamp.org/variant.html?variant=rs9909004) | PR interval |  | 2.86E-09 |
| ATP2B1 | [rs882781](https://cvd.hugeamp.org/variant.html?variant=rs882781) | LDL cholesterol | Lipids | 1.52E-16 |
|  | [rs7313499](https://cvd.hugeamp.org/variant.html?variant=rs7313499) | Total cholesterol |  | 6.93E-13 |
|  | [rs2230283](https://cvd.hugeamp.org/variant.html?variant=rs2230283) | Non-HDL cholesterol |  | 2.3E-11 |
|  | [rs2681472](https://cvd.hugeamp.org/variant.html?variant=rs2681472) | Systolic blood pressure | Cardiovascular | 4.77E-128 |
|  | [rs2681472](https://cvd.hugeamp.org/variant.html?variant=rs2681472) | Pulse pressure |  | 3.38E-92 |
|  | [rs17249754](https://cvd.hugeamp.org/variant.html?variant=rs17249754) | Diastolic blood pressure |  | 6.29E-75 |
|  | [rs2681485](https://cvd.hugeamp.org/variant.html?variant=rs2681485) | Hypertension |  | 6.03E-39 |
|  | [rs1401982](https://cvd.hugeamp.org/variant.html?variant=rs1401982) | Mean arterial pressure |  | 4.43E-19 |
|  | [rs2681492](https://cvd.hugeamp.org/variant.html?variant=rs2681492) | Coronary artery disease |  | 1.9E-14 |
|  | [rs7302816](https://cvd.hugeamp.org/variant.html?variant=rs7302816) | Ascending aorta diameter |  | 0.000000025 |
|  | [rs2408058](https://cvd.hugeamp.org/variant.html?variant=rs2408058) | Height | Anthropometric | 2.84E-15 |
|  | [rs12298263](https://cvd.hugeamp.org/variant.html?variant=rs12298263) | BMI |  | 1.42E-10 |
| PIK3R3 | [rs113046963](https://cvd.hugeamp.org/variant.html?variant=rs113046963) | LDL cholesterol | Lipids | 9.98E-15 |
|  | [rs61782881](https://cvd.hugeamp.org/variant.html?variant=rs61782881) | Total cholesterol |  | 5.24E-12 |
|  | [rs3845301](https://cvd.hugeamp.org/variant.html?variant=rs3845301) | Non-HDL cholesterol |  | 6.95E-09 |
|  | [rs1707336](https://cvd.hugeamp.org/variant.html?variant=rs1707336) | HDL cholesterol |  | 4.16E-08 |
|  | [rs11576668](https://cvd.hugeamp.org/variant.html?variant=rs11576668) | Heart rate | Cardiovascular | 4.98E-15 |
|  | [rs12143773](https://cvd.hugeamp.org/variant.html?variant=rs12143773) | Diastolic blood pressure |  | 1.65E-11 |
|  | [rs11211199](https://cvd.hugeamp.org/variant.html?variant=rs11211199) | Pulse pressure |  | 1.17E-10 |
|  | [rs12143448](https://cvd.hugeamp.org/variant.html?variant=rs12143448) | Hypertension |  | 8.17E-09 |
|  | [rs2230657](https://cvd.hugeamp.org/variant.html?variant=rs2230657) | Height | Anthropometric | 4.33E-29 |
|  | [rs1707322](https://cvd.hugeamp.org/variant.html?variant=rs1707322) | BMI |  | 1.74E-14 |
|  | [rs811485](https://cvd.hugeamp.org/variant.html?variant=rs811485) | Waist-hip ratio |  | 4.67E-08 |
| PTCH1 | [rs357564](https://cvd.hugeamp.org/variant.html?variant=rs357564) | Height | Anthropometric | 4.42E-130 |
|  | [rs473902](https://cvd.hugeamp.org/variant.html?variant=rs473902) | Hip circumference adj BMI |  | 8.38E-15 |
|  | [rs473902](https://cvd.hugeamp.org/variant.html?variant=rs473902) | Waist circumference adj BMI |  | 1.76E-13 |
|  | [rs7854560](https://cvd.hugeamp.org/variant.html?variant=rs7854560) | Waist circumference adj BMI-smoking status |  | 5.71E-12 |
|  | [rs7854560](https://cvd.hugeamp.org/variant.html?variant=rs7854560) | Waist-hip ratio adj BMI |  | 6.03E-11 |
|  | [rs2810915](https://cvd.hugeamp.org/variant.html?variant=rs2810915) | Atrial fibrillation |  | 7.33E-12 |
| IL6 | [rs2069827](https://cvd.hugeamp.org/variant.html?variant=rs2069827) | HDL cholesterol | Lipids | 1.74E-11 |
|  | [rs1800795](https://cvd.hugeamp.org/variant.html?variant=rs1800795) | Pulse pressure | Cardiovascular | 2.73E-19 |
|  | [rs13242809](https://cvd.hugeamp.org/variant.html?variant=rs13242809) | Plasma C-reactive protein |  | 4.17E-17 |
|  | [rs1800797](https://cvd.hugeamp.org/variant.html?variant=rs1800797) | Systolic blood pressure |  | 5.24E-11 |
