# Supplementary figures and images for: Prioritization of microRNA biomarkers for a prospective evaluation in a cohort of myocardial infarction patients based on their mechanistic role using public datasets
Source: Front Cardiovasc Med. 2022 Nov 3;9:981335. doi: 10.3389/fcvm.2022.981335 (PMC9668885; doi:10.3389/fcvm.2022.981335)

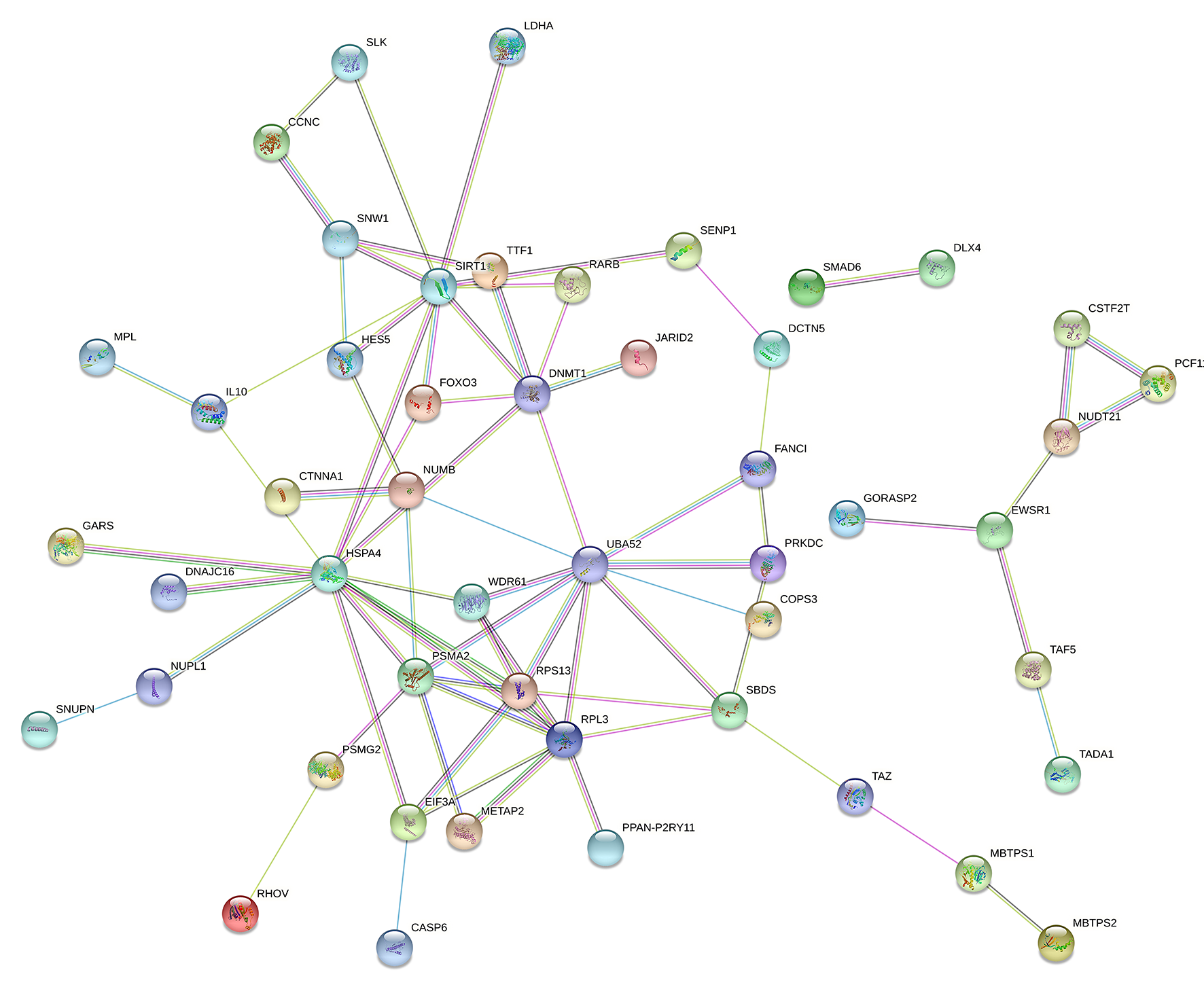

Supplement: Supplementary Figure 1 — A representative image of the PPI interaction network for the predicted target genes. Colored nodes (circles) indicate direct/first line interactions among the genes. Purple and blue color lines denote known interactions determined experimentally and from curated databases, respectively. Black, dark green and light green lines indicate co-expression, neighborhood gene and interactions derived from text mining, respectively. Thickness of the lines indicates strength of data support. [file Image_1.TIF]

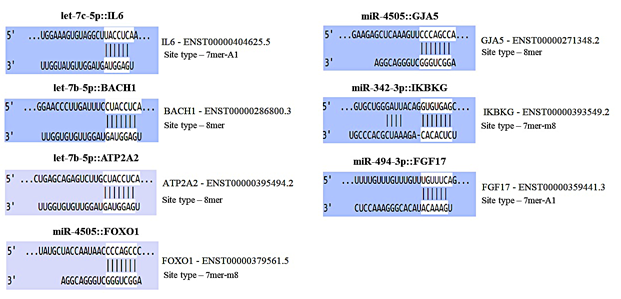

Supplement: Supplementary Figure 2 — MicroRNA binding sites on target genes for prioritized miR candidates obtained using TargetScan Human 7.2 database. [file Image_2.TIF]

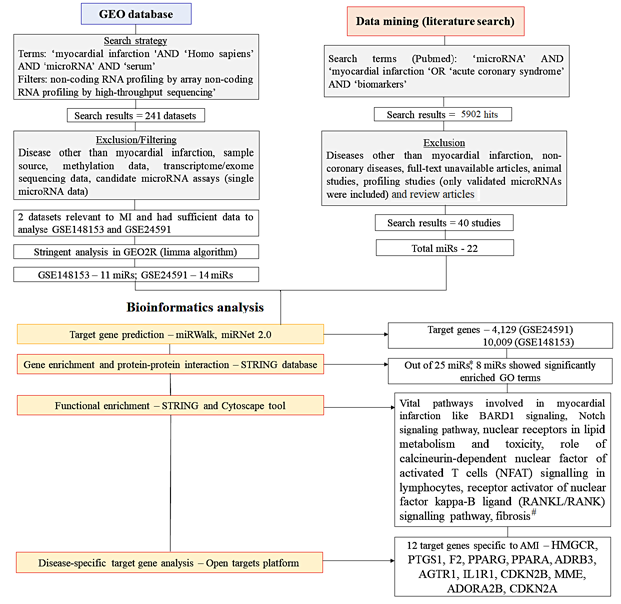

Supplement: Supplementary Figure 3 — Flow chart illustrating the summary protocol and findings of in silico analysis using bioinformatics tools. “*” Denotes 25 miRs obtained from GSE148153 and GSE24591 datasets only; “#” denotes partial list of the vital functions/pathways involved in myocardial infarction. [file Image_3.TIF]
